# Two high-quality *Prototheca zopfii* genomes provide new insights into their evolution as obligate algal heterotrophs and their pathogenicity

**DOI:** 10.1128/spectrum.04148-23

**Published:** 2024-06-28

**Authors:** Jianbo Jian, Zhaoyang Wang, Chunhai Chen, Christopher T. Workman, Xiaodong Fang, Thomas Ostenfeld Larsen, Jian Guo, Eva C. Sonnenschein

**Affiliations:** 1 Department of Biotechnology and Biomedicine, Technical University of Denmark, Lyngby, Denmark; 2 BGI Genomics, Shenzhen, China; 3 Guangdong Provincial Key Laboratory of Marine Biotechnology, Shantou University, Shantou, China; 4 Department of Laboratory Medicine, Shanghai East Hospital, School of Life Sciences and Technology, Tongji University, Shanghai, China; 5 Department of Biosciences, Swansea University, Swansea, United Kingdom; University of Mississippi, University, Mississippi, USA

**Keywords:** *Prototheca*, pathogenicity, horizontal gene transfer, heterotrophs

## Abstract

The majority of the nearly 10,000 described species of green algae are photoautotrophs; however, some species have lost their ability to photosynthesize and become obligate heterotrophs that rely on parasitism for survival. Two high-quality genomes of the heterotrophic algae *Prototheca zopfii* Pz20 and Pz23 were obtained using short- and long-read genomic as well as transcriptomic data. The genome sizes were 31.2 Mb and 31.3 Mb, respectively, and contig N50 values of 1.99 Mb and 1.26 Mb. Although *P. zopfii* maintained its plastid genome, the transition to heterotrophy led to a reduction in both plastid and nuclear genome size, including the loss of photosynthesis-related genes from both the nuclear and plastid genomes and the elimination of genes encoding for carotenoid oxygenase and pheophorbide an oxygenase. The loss of genes, including basic leucine-zipper (bZIP) transcription factors, flavin adenine dinucleotide-linked oxidase, and helicase, could have played a role in the transmission of autotrophy to heterotrophs and in the processes of abiotic stress resistance and pathogenicity. A total of 66 (1.37%) and 73 (1.49%) genes were identified as potential horizontal gene transfer events in the two *P. zopfii* genomes, respectively. Genes for malate synthase and isocitrate lyase, which are horizontally transferred from bacteria, may play a pivotal role in carbon and nitrogen metabolism as well as the pathogenicity of *Prototheca* and non-photosynthetic organisms. The two high-quality *P. zopfii* genomes provide new insights into their evolution as obligate heterotrophs and pathogenicity.

**IMPORTANCE:**

The genus *Prototheca*, characterized by its heterotrophic nature and pathogenicity, serves as an exemplary model for investigating pathobiology. The limited understanding of the protothecosis infectious disease is attributed to the lack of genomic resources. Using HiFi long-read sequencing, both nuclear and plastid genomes were generated for two strains of *P. zopfii*. The findings revealed a concurrent reduction in both plastid and nuclear genome size, accompanied by the loss of genes associated with photosynthesis, carotenoid oxygenase, basic leucine-zipper (bZIP) transcription factors, and others. The analysis of horizontal gene transfer revealed the presence of 1.37% and 1.49% bacterial genes, including malate synthase and isocitrate lyase, which play crucial roles in carbon and nitrogen metabolism, as well as pathogenicity and obligate heterotrophy. The two high-quality *P. zopfii* genomes represent valuable resources for investigating their adaptation and evolution as obligate heterotrophs, as well as for developing future prevention and treatment strategies against protothecosis.

## INTRODUCTION

Autotrophic eukaryotes have emerged from multiple independent endosymbiotic events involving photosynthetic organisms (1, 2). Despite the significant benefits of photosynthesis, secondary losses of this process have occurred independently in various algae and plants (3). Algae are highly diverse aquatic organisms that can be classified based on their nutritional mode, including free-living or symbiotic autotrophy, mixotrophy, and heterotrophy (4). The free-living autotrophic or symbiotic algae are capable of self-sustenance and synthesize their organic compounds *via* photosynthesis, while mixotrophic algae exhibit greater flexibility by utilizing both photosynthesis and other organic carbon sources for energy and nutrient acquisition (4). Obligate heterotrophic or parasitic algae, in contrast, must obtain their energy and nutrients from other organisms or a host within their environment.

*Prototheca* species, which belong to the Chlorellaceae family of green algae, are widely distributed in natural organic materials around the world (5). Unlike photosynthetic algae, they have forfeited the photosynthetic ability to be obligate heterotrophic algae and switched to parasitism (6, 7). Due to their yeast-like colony morphology, these organisms were once even classified as fungi (8). *Prototheca* have been identified as causative pathogens of human and animal disease, which not only impacts the human health but also result in significant economic losses in livestock (9). *Prototheca* biofilms resemble those formed by pathogenic bacteria and fungi, leading to reduced immune cell activation and increased resistance to antimicrobials. This contributes to the development of chronic and difficult-to-treat infections (10).

To date, the reason behind *Prototheca*’s transition from photosynthetic ability to obligate heterotrophy, and parasitism, remains unclear due to insufficient genomic data and limited knowledge of this organism. However, with the recent advancements in high-throughput sequencing, including both short-read and long-read sequencing, coupled with a decrease in cost over the years, several *Prototheca* organelle and nuclear genomes have been published or made available. A total of five complete plastid genomes were available in NCBI as of 4th May 2023, comprising one each for *Prototheca cutis*, *Prototheca stagnorum*, *Prototheca bovis*, *Prototheca ciferrii,* and *Prototheca wickerhamii*. The size of the *P. wickerhamii* plastid genome (55,636 bp) was slightly smaller than that of the closely related photosynthetic *Auxenochlorella protothecoides* (84,580 bp), which resulted from the loss of photosynthetic genes after their evolutionary divergence (11). In addition, the mitochondrial (~38 Kb) and plastid (~28 Kb) genomes of *P. ciferrii* and *P. bovis* were among the smallest known in the Trebouxiophytes; all photosynthesis-related genes were lost in their plastid genomes (7). The mitochondrial genome of the human pathogen *P. wickerhamii* is 53.8 kb while its plastid genome is 48 kb; both contain similar gene content to other *Prototheca* species (12, 13). A total of eight *Prototheca* nuclear genome sequences are reported or available in the NCBI database to date, including five genomes based on short-read sequencing data: *P. ciferrii* SAG2063 (7), *Prototheca cutis* JCM 15793 (6), *P. cutis* 20–25310, *Prototheca stagnora* JCM 9641 (6), *P. bovis* SAG 2021 (7), and three genomes based on long-read sequencing data: *P. wickerhamii* strains ATCC 16529 (14), *P. wickerhamii* strains S1 and S931 (13). The genome of *P. wickerhamii* was found to be compacted and highly similar to that of non-pathogenic closely related *A. protothecoides*, exhibiting a loss of certain genes associated with photosynthesis but containing numerous enzymes and genes related to pathogenicity (13, 14). Multiple independent losses of photosynthesis have been identified through phylogenetic analyses based on plastid and nuclear genome proteins (6).

HGT (Horizontal gene transfer) is a prevalent mechanism for adaptation in prokaryotes, including bacteria and archaea. The acquisition of antibiotic resistance and pathogenicity by microorganisms is often attributed to HGT (15). HGT events are infrequent in eukaryotes, including algae; however, they could confer adaptive advantages and play a crucial role in major lifestyle transitions (16). HGT events have been found to confer new functions from prokaryotic donors to algae in various extreme environments, including polar climates (17), high temperatures (18), salinity, and other stresses (19). Recent research suggests that HGT events have played a crucial role in facilitating the evolution and adaptation of charophyte green algae as well as land plants (20–23). For example, 3%–5% of diatom genes have a horizontal origin that could contribute to their ecological importance and expand their adaptive capabilities (24). Relevant studies have previously reported the acquisition of pathogenic properties through HGT (25, 26).

*P. zopfii* is a unicellular, achlorophyllous microalgae ubiquitous in nature and is an opportunistic, environmental pathogen (27). *P. zopfii* and *P. wickerhamii* are the most common pathogenic species of the *Prototheca* genus (10). *P. zopfii* was initially identified as the etiological agent of bovine mastitis in Germany in 1952 (28, 29). A total of over a dozen countries have reported cases of prototheca mastitis, resulting in significant economic losses (30, 31). A previous study documented the organelle and nuclear genomes of *P. zopfii* genotype 1 and genotype 2, which have since been reclassified as *P. ciferrii* and *P. bovis*, respectively (7, 32). The short-read-based assembly of three nuclear genomes of *P. zopfii* was utilized to augment the virulent capacity of pathogenic strains; however, fully assembled genomes were not publicly available (33). By integration of publicly available *Prototheca* genomes and two herein presented high-quality, long-read-based *P. zopfii* genomes, we aim to contribute to the role of a *Prototheca* in pathogenesis and its evolutionary adaptation.

## RESULTS AND DISCUSSION

### Genome analysis of *P. zopfii*

**Fig 1:**
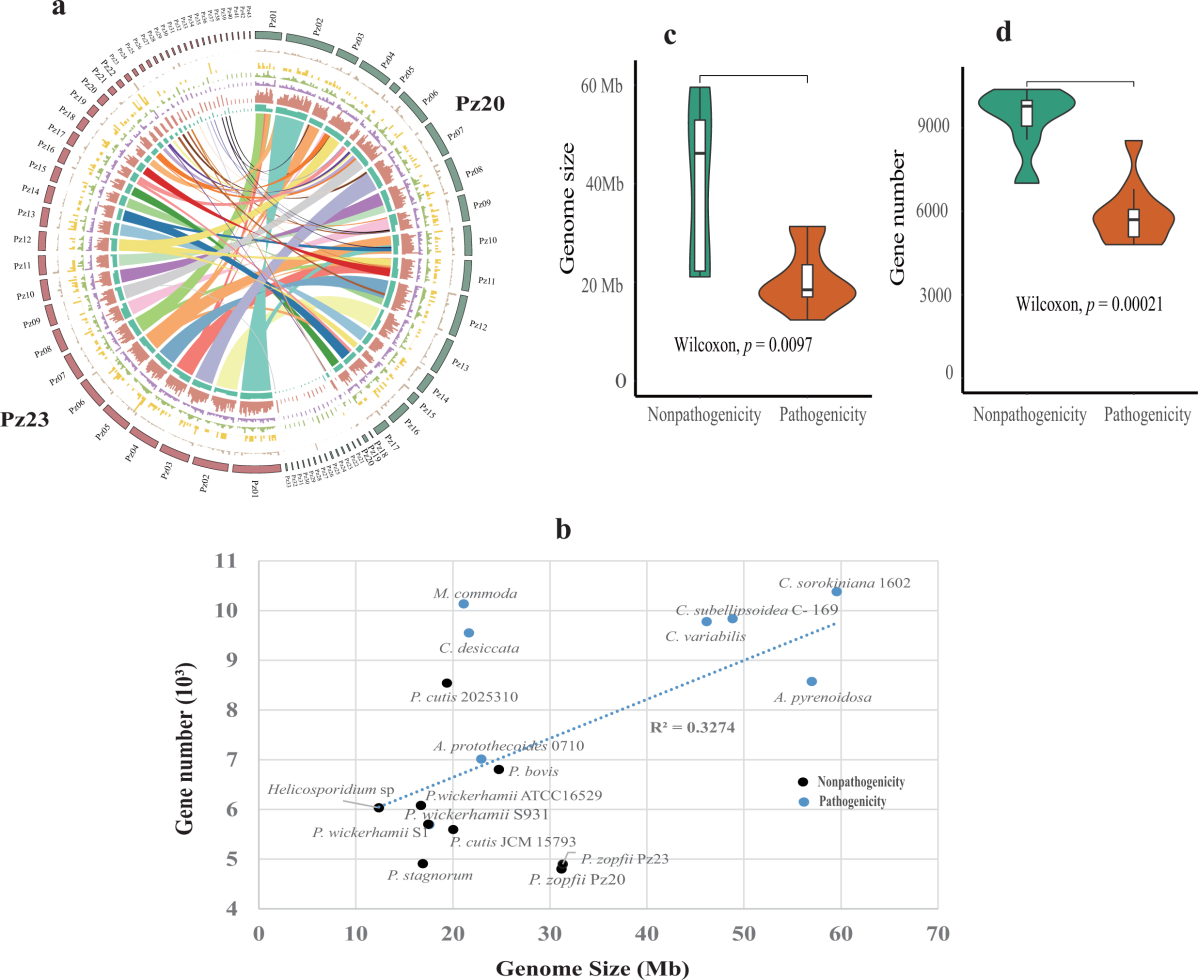
The genome characteristic of *Prototheca zopfii* and comparison with other sequenced green algae. (a) The circus plot of two *P. zopfii* strains. (Gene collinearity, GC content, gene density, transposons, DNA repeats, copia, and gypsy elements). The left brown is Pz23 and the right blue is Pz20. (b) The relationship of genome size and gene number in the available and newly sequenced Trebouxiophyceae genomes. (c and d) The violin plot of genome size and gene number comparison between pathogenicity and non-pathogenicity with Wilcoxon test.

To present high-quality genomes of *P. zopfii*, first the optimal sequencing depth for long-read strategies was determined, and genome size and heterozygosity for *P. zopfii* Pz20 were estimated using short-read data. After filtering out low-quality reads, a total of 4.56 Gb of clean DNBSeq short-read data were generated for *P. zopfii* Pz20. The Q20 and Q30 values of the data, which were 96.49% and 91.76%, respectively (Table S1). The diploid nature of *P. zopfii* Pz20 was determined based on the frequency analysis of the jellyfish 21-mer, revealing an estimated haploid genome size of approximately 28.5 Mb and a heterozygosity rate of 1.4%, as indicated by the genomeScope profile (Fig. S1). Subsequently, a total of 4.12 Gb and 3.14 Gb long-read data were generated for *P. zopfii* Pz20 and *P. zopfii* Pz23, respectively, using the PacBio circular consensus sequencing (CCS) model. Both the average read length and N50 length exceeded 17 kb (Table S2). The HiFi reads were assembled using Hifiasm, resulting in 31.2 Mb and 31.3 Mb genome sequences for Pz20 and Pz23 after removing the organelle genomes, respectively (Table 1; Table S3). Among the *Prototheca* genus, *P. zopfii* exhibits the largest genome size (7). The contig N50 values for Pz20 and Pz23 were 1,987,055 bp (74 contigs) and 1,261,121 bp (80 contigs), respectively (Table 1; Fig. 1a). The contig N50 and a number of contigs are greater or lesser than those of the genomes of *P. wickerhamii* S1, S931, and ATCC 16529 (13, 14), respectively, but significantly higher than those of other *Prototheca* species including *P. bovis* (GCA_003612995.1), *P. cutis* JCM 15793 (GCA_002897115.2), and *P. stagnorum* (GCA_002794665.1) with genome sizes ranging from 16.9 Mb to 24.7 Mb (Table 1) (6, 7, 13). The GC content of *P. zopfii* is relatively high, with values of 67.7% and 67.8%, which are slightly lower than those observed in *P. bovis* (73.5%) and *P. stagnorum* (71.4%), but slightly higher than observed in *P. wickerhamii* (~64%) and higher than the range observed in other algae genomes (44.9%–64.45%) (Table S4). 78.3% and 78.4% of genes were deemed complete in the Pz20 and Pz23 genomes, respectively, according to BUSCO V5 analysis based on chlorophyta_odb10 (a total of 1519 gene set) (Table S5) and comparable with other *Prototheca* species (7, 13) .

**TABLE 1:** The genomic features of two newly strains of *P. zopfii* and four available *Prototheca* species

Species	*P. zopfii* Pz20 (this study)	*P. zopfii* Pz23 (this study)	*P. bovis* (GCA_003612995.1)	*P. cutis* JCM 15793 (GCA_002897115.2）	*P. stagnorum* (GCA_002794665.1)	*P. wickerhamii* S1 (Guo, J., et al. 2022)
Assembled genome size (Mb)	31,193,587	31,323,842	24,744,895	20,029,045	16,896,228	17,573,978
Sequencing technologies	PacBio Sequel II	PacBio Sequel II	Illumina MiSeq; 454	HiSeq 2500	HiSeq 2500	Nanopore PromethION
No of scaffold	74	80	4,555	46	27	19
Scaffold N50	1,987,055	1,261,121	7,940	1,409,608	1,107,247	1,639,047
No of contig	74	80	4,555	650	853	19
Contig N50	1,987,055	1,261,121	7,940	56,125	33,265	1,639,047
GC content	67.70%	67.80%	73.53%	60.24%	71.40%	64.21%
Max length	2,960,522	2,950,851	57,068	2,465,851	1,567,793	2,552,045
No of genes	4,801	4,899	6,805	5,599	4,915	5,694
Complete BUSCOs (Gene)	77.3%	77.6%	75.8%	77.4%	75.5%	88.4%

### Comparison of plastid genomes

**Fig 2:**
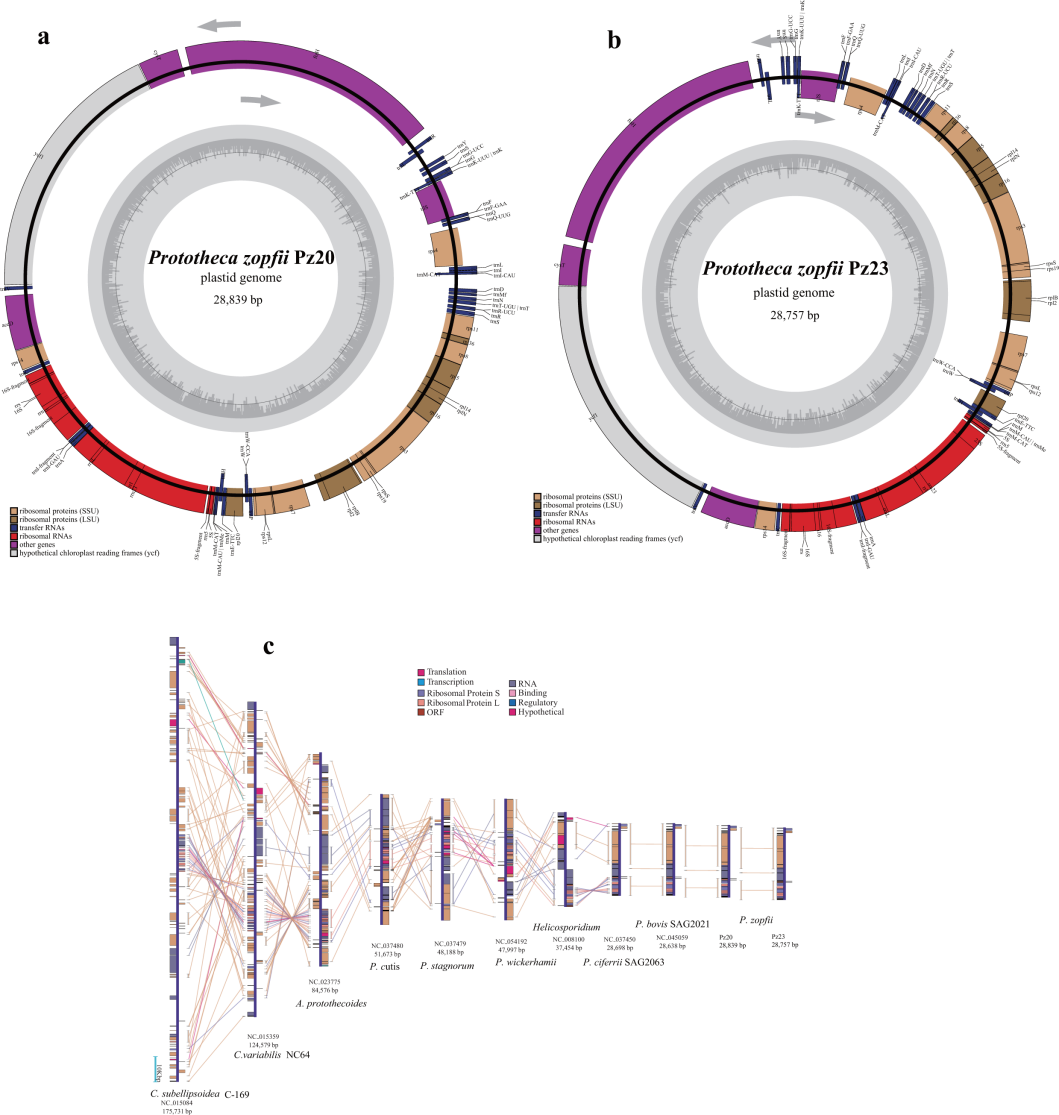
The plastid genome characteristic of *Prototheca zopfii* and gene collinearity. (a) The plastid genome map of *P. zopfii* Pz20. (b) The plastid genome map of *P. zopfii* Pz23. (c) Gene order comparisons among the newly sequenced plastid genomes of Pz20, Pz23, and other closely related Chlorellaceae species. The linear represents the conserved gene collinearity.

Through endosymbiosis with photosynthetic symbionts, eukaryotes acquired plastids which provide benefits to primary production (1). All *Prototheca* species possess colorless plastids and undergo a transition from photosynthetic to heterotrophic lifestyle (6). The plastid genomes of Pz20 and Pz23 were each assembled into one circular contig, with a length of 28,839 bp and 28,757 bp, respectively (Fig. 2a and b; Table S6). The plastid genomes of *P. ciferrii* SAG2063, *P. bovis* SAG 2021, Pz20 and Pz23, were found to be the smallest among closely related Trebouxiophyceae plastid genomes, ranging from 37,454 bp to 51,673 bp (Fig. 2c). Both plastid genomes exhibited a relatively low GC content, with Pz20 and Pz23 containing 26.98% and 26.78%, respectively. Both *P. zopfii* strains were predicted to have 23 protein-coding genes, and their gene composition was found to be like that of *P. ciferrii* SAG2063 and *P. bovis* SAG2021 (Table S6). Conserved gene collinearity was observed among *Coccomyxa subellipsoidea* C-169, *Chlorella variabilis* NC64, and *Auxenochlorella protothecoides* despite differences in plastid genome size, without loss of genes detected in the photosynthetic algae (Fig. 2c). All *Prototheca* species and the colorless, pathogenic *Helicosporidium* lacked genes related to photosynthesis and ATP, except for *P. cutis* which retained six ATP synthase genes (atpA, atpB, atpE, atpF, atpH, and atpI), as well as an ATP-dependent gene (clpP) like that found in *P. wickerhamii* (Fig. 2c). Thus, the non-photosynthetic, obligate heterotrophic species possess a compact plastid genome devoid of functional genes for photosynthesis and ATP production (6, 11, 34). The analogous phenomenon had also been documented in a non-parasitic secondary heterotroph, diatom *Nitzschia putrida* (35). The transition from a free-living to a heterotrophic lifestyle implies that algae adopting parasitic lifestyles require specific adaptations to their host environments, which subsequently leads to the dispensability and subsequent loss of genes related to photosynthesis and ATP synthase.

### Comparison of repeat and gene features

By combining homology-based and *de novo* methods, we identified 2.88 Mb (12.4%) and 3.94 Mb (12.6%) of repetitive sequences in Pz20 and Pz23, respectively (Tables S7 and S8). LTRs (long-terminal repeats) were found to be the most abundant element, accounting for 7.5% and 8.3% of the whole genome in Pz20 and Pz23, respectively (Fig. S3; Tables S7 and S8). The proportion of repeats in the *P. zopfii* genome is a slightly higher than that in the *C. variabilis* NC64A genome (12%) (36), but significantly higher than that in the *P. wickerhamii* genomes (3.1% and 2.5%) (13). The 1.5-fold difference in genome size between *P. zopfii* and *P. wickerhamii* can be attributed to the presence of repetitive sequences, which were previously considered as “junk” DNA but may actually have important roles in evolution or biological functions (37).

A total of 4,801 and 4,899 protein-coding genes were annotated for Pz20 and Pz23 using the MAKER pipeline (Table 1). The relationship between genome size and gene number in the 16 Trebouxiophyceae genomes and *M. commoda* was found to be linear with an R^2^ value of 0.33 (Fig. 1b). The pathogenic (non-photosynthetic heterotrophic) species, including those of the *Prototheca* genus and *Helicosporidium* sp., exhibit smaller genome sizes and fewer genes overall, except for *A. protothecoides* 0710 (Fig. 1b). The genome size and gene number of pathogenicity are significantly lower (*P* < 0.01) than those of non-pathogenicity (autotrophy) (Fig. 1c and d). The findings indicate that lifestyle transition led to a reduction in genome size and gene loss. The quality of the gene annotation was evaluated for all the target 17 green algae. 77.3% and 77.6 % of genes were evaluated to be complete for the predicted genes in Pz20 and Pz23, respectively (Fig. S4). The BUSCO completeness score is slightly higher than that of *P. bovis*, *P. cutis*, *P. stagnorum* but smaller than that of *P. wickerhamii* (Fig. S4). The BUSCO completeness scores of non-photosynthetic algae generally exhibit lower values than those of photosynthetic algae, indicating the potential loss of conserved nuclear genes in addition to plastid genes.

With a total of seven databases, including NR, Swiss-prot, KEGG, KOG, TrEMBL, Interpro, and GO, the protein-coding genes were functionally annotated (38–40). The KOG annotation of both Pz20 and Pz23 showed that most genes were related to “general function prediction only” and posttranslational modification, protein turnover, chaperones, followed by translation, ribosomal structure, and biogenesis (Fig. S5 and S6). A high number of genes were enriched in metabolic pathways according to the KEGG enrichment analysis (Fig. S7 and S8). The two strains encoded a total of 285 and 288 genes, with the majority being involved in carbohydrate metabolism, which is central to numerous essential metabolic pathways (Fig. S7 and S8). The number of genes associated with amino acid and lipid metabolism was also found at the same level in both genomes. In both strains, 42 and 38 genes were enriched in the metabolism of terpenoids and polyketides, slightly more than found in *P. wickerhamii* (~30) (13). A total of 40 and 37 genes potentially associated with environmental adaptation were identified in Pz20 and Pz23, respectively (Fig. S7 and S8), both of which are smaller than in autotrophic algae (Table S9). The evolution of obligate heterotrophs and pathogenicity resulting from environmental adaptation strategies is a crucial aspect in comprehending ecological functions. For Pz20 and Pz23, 98.7% and 98.6% of genes were functionally annotated in the seven databases, respectively (Table S10). The high percentage of annotation is consistent with that observed in *P. wickerhamii* (>95%), indicating that most *Prototheca* genes are not novel or unique to this genus (13, 14).

**Fig 3:**
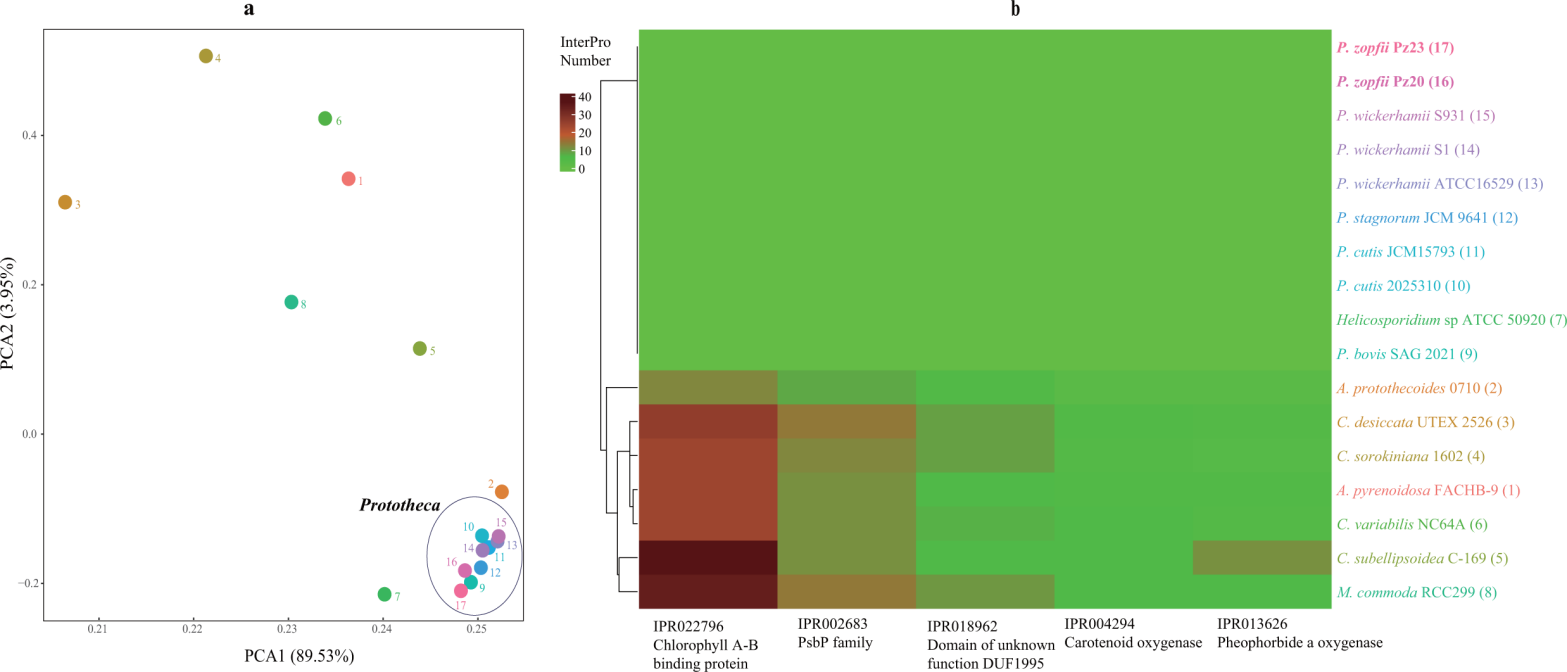
The PCA and heatmap analysis InterPro in 17 algae genomes. (a) The PCA analysis of the top 10% of the number of InterPro. (b) The heatmap of five InterPro domains that are lost in the genus *Prototheca*. The various species of a with number, represented by b in brackets.

Functional diversity patterns were investigated using principal component analysis (PCA) and heatmap, based on InterPro (IPR) scanning utilizing Pfam domains. The PCA results of IPR domains, which identified the top 10% of conserved protein domains, revealed that all *Prototheca* species formed a distinct cluster separate from other algae among the 17 analyzed green algal species (Fig. 3a). The heatmap depicting the top 10% of IPR numbers revealed that *Prototheca* formed a cluster near *A. protothecoides* 0710 (Fig. S9), thereby corroborating the results obtained from the PCA analysis. The IPR patterns suggested that *Prototheca* and other closely related algae had undergone significant functional diversification. Interestingly, five IPR domains (IPR022796, IPR002683, IPR018962, IPR004294, and IPR01362) were found to be completely absent in all the nine *Prototheca* species and *Helicosporidium* sp, whereas these domains were present in seven other photosynthetic algae species (Fig. 3b). Chlorophyll A-B binding protein (IPR022796) is the apo-protein of the light-harvesting complex of photosystem (PS) II (41) and the PsbP family (IPR002683) is required for PSII (42). Both nuclear-encoded IPR genes were lost simultaneously with the plastid photosynthesis-related genes, implying that the gene loss occurred not only in the plastid but also in the nuclear genome. For phototrophic organisms, carotenoids are also essential for the process of photosynthesis in addition to chlorophylls. Therefore, carotenoid oxygenase (IPR004294) was eliminated convergently. Pheophorbide an oxygenase (IPR013626) is a Rieske-type iron-sulfur protein (43) serving as a regulator of chlorophyll catabolism. Due to the loss of the chlorophyll genes, the catabolism gene has also become non-functional and lost during evolution. The functional role of the DUF1995 domain (IPR018962) remains unknown, but it can be found in a chloroplastic protein (44) and its absence suggests a potential involvement in photosynthesis. No significantly enriched IPR domains were observed for *P. zopfii* Pz20 and Pz23 when compared to all other non-pathogenic species, which conflicts with the findings of a previous study that only compared a single *P. wickerhamii* ATCC16529 genome to another (13). A less total number of IPR domains was commonly observed compared with non-pathogenic species. In *P. zopfii*, 12 IPRs were found to be enriched (>3 domains) with an average when compared to other *Prototheca* species (Table S11). These genes, including FAD-linked oxidase (which functions in defense) and helicase (which functions in DNA replication), may contribute to the diversity of *P. zopfii*.

### Gene family and phylogenetic analysis

A total number of 124,335 predicted protein genes from all 17 algal genomes were clustered into 10,119 orthogroups using OrthoFinder software (Table S12). The number of gene orthogroups that were clustered within a species varied from 4,646 (*P. stagnorum*) to 9,678 (*C. sorokiniana*) (Table S12). There was a total of 1,155 species-specific orthogroups identified among the 17 genomes. A total of 258 single-copy orthologous gene families were identified and utilized for the construction of the phylogenetic tree with *M. commoda* as the outgroup. All *Prototheca* genomes were classified into the same clade as *A. protothecoides* 0710 and *Helicosporidium* sp. (Fig. 3). However, *A. pyrenoidosa* was found to belong to the same clade as the *Chlorella* genus rather than being grouped with *A. protothecoides. A. pyrenoidosa* has been previously classified as *Chlorella pyrenoidosa* (45). The divergence of *Prototheca* from *Chlorella* occurred approximately 618 million years ago (Mya). The two strains of *P. zopfii* exhibited the closest phylogenetic relationship with *P. bovis* and diverged approximately 173.6 Mya (Fig. 3). The phylogenetic analysis yielded results consistent with those of the previous study (13). The gene synteny indicates a high degree of gene block alignment, although only a few genes are syntenic between different *Prototheca* species (Fig. S10). The inter-comparison shows a positive correlation of gene conservation with the phylogenetic relationship. The genome size and the absence of photosynthesis-related genes vary among *Prototheca* species. The *Prototheca* species have undergone long-term divergence, indicating that speciation and independent evolution with the loss of photosynthesis occurred multiple times within the clade of *Prototheca* (6). The analysis of gene expansions and contractions was employed to discern the diversity and adaptation across various clades or species. A total of 558 and 98 gene families exhibited contractions in the two sub-clades of heterotrophs, while only a few families showed expansions, including *Helicosporidium* sp., *P. bovis* and *P. zopfii* and *P. stagnorum* or *A. protothecoides*, *P. cutis,* and *P. wickerhamii* on the other hand, thus confirming the occurrence of gene loss. When comparing to the photosynthetic algae, only four gene families experienced expansion while 739 gene families underwent contraction in the clade of the heterotrophs. No significantly expanded genes (*P* < 0.01) were observed. For the contracted genes, the GO enrichment involved isomerase activity and protein kinase activity, both of which have also experienced a reduction in the number of InterPro domains (Table S13). Also, the plant hormone signal transduction pathway and the MAPK signaling pathway exhibited significant involvement in the contracted genes. Plant hormones regulate the response of plants to abiotic stresses, thereby influencing plant growth and survival (46). The MAPK signaling pathway is also involved in the plant’s ability to defend against pathogens (47). In the *P. zopfii* branch, a total of 58 and 480 gene families underwent expansion and contraction, respectively. The genes that have significantly expanded (*P* < 0.01) were notably enriched in the metabolism of ascorbate and aldarate, as well as the biosynthesis of secondary metabolites (Table S13). As one of the crucial pathways involved in carbohydrate metabolism, the ascorbate and aldarate metabolic pathway can protect cells against oxidative damage caused by aerobic metabolism and pathogenic infections (48). The expansion of biosynthetic pathways for secondary metabolites in *P. zopfii* suggests their involvement in virulence mechanisms and infection processes (49, 50). The contracted genes were enriched in biotin metabolism and ABC (ATP-binding cassette) transporters showed that some related function lost during evolution (51).

### The loss of regulated genes and putatively pathogenicity

**Fig 4:**
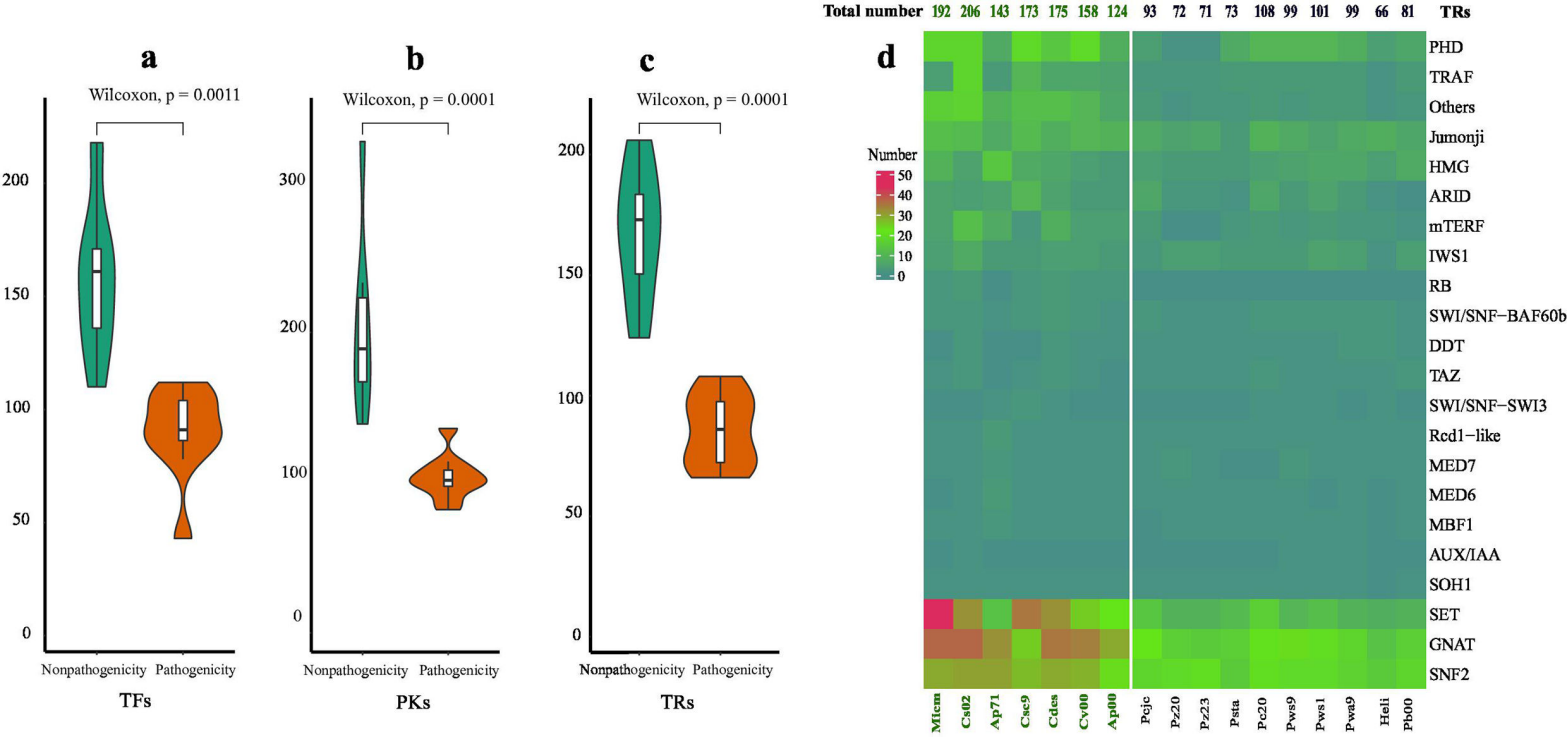
The gene lost comparison between pathogenicity and non-pathogenicity and heatmap of TRs number. (a) The violin plot of comparison between pathogenicity and non-pathogenicity with Wilcoxon test for TFs (transcription factors). (b) The violin plot of comparison between pathogenicity and non-pathogenicity with Wilcoxon test for TRs (transcriptional regulators). (c) The violin plot of comparison between pathogenicity and non-pathogenicity with Wilcoxon test for PKs (protein kinases). (d) The heatmap of TRs number. The strains of abbreviation can be found in Table S12.

The TFs (transcription factors), TRs (transcriptional regulators), and PKs (protein kinases) are essential in regulating gene expression, regulation, and cellular signaling. The iTAK pipeline was used to identify the patterns of TFs, TRs, and PKs to compare autotrophs and heterotrophs. The number of genes of TFs, TRs, and PKs were significantly different between autotrophs and heterotrophs (Wilcox test, *P* < 0.001) (Fig. 4a through c). This suggests that not only the nuclear photosynthesis-related genes were lost but also their regulatory genes. Transcription factors (TFs) regulate the transcription of genetic information from DNA to messenger RNA, and they are essential for the precise regulation of gene expression (52). The iTAK pipeline (53) identified a total number of TFs, indicating that heterotrophic species (43 to 112 TFs) have significantly lower numbers compared to other autotrophic species (110 to 218 TFs) (Fig. S11). The loss of photosynthesis-related genes may occur concomitantly with a decrease in the abundance of TFs, whereas *P. cutis* 2025310 with 112 TFs suggests only partial loss. The difference in average values between *Prototheca* and autotrophic algal species, which exceeds 3 (Fig. S11), indicates these TFs were reduced in *Prototheca* including SBP (SQUAMOSA promoter-binding protein), C3H (Cysteine3Histidine), bZIP (basic leucine zipper), C2H2 (Cys2-His2), MYB and MYB-related family. All these TFs belong to super families studied in plants to be involved in developmental processes, growth, and environmental responses. The loss of these TFs family suggests that the transmission from autotrophy to heterotrophs is associated with the process of developing abiotic stress resistance and may also be important in pathogenicity. The bZIP transcription factors regulate processes such as pathogen defense, light signaling, and stress response (54).

The comparison of autotrophic and heterotrophic algae reveals distinct differences in three types of TRs, namely SET (Su(var)3–9, Enhancer-of-zeste, Trithorax), GNAT (GCN5-related N-acetyltransferase), and SNF2 (Sucrose nonfermenting 2) (Fig. 4e). The epigenetic marker SET has been identified as a key regulator of chromatin-mediated gene transcription. SET may participate in the biological process of manipulating host transcription to establish infection (55). The role of the SET protein in host-pathogen interactions may be response the pathogenicity in *Prototheca*. GNAT superfamily is widely distributed and plays a critical role in various cellular processes, ranging from antibiotic resistance to histone modification (56). SNF2 proteins play a crucial role in epigenetic regulation, serving as key modulators of gene expression and genome stability (57) and has been identified as a biocontrol regulator that governs antifungal activity (58).

To investigate the pathogenicity of *Prototheca* genomes, we utilized Blastp with diamond to conduct a search in the Pathogen Host Interaction (PHI) database (59). A total of 1,160 and 1,154 protein-coding genes of Pz20 and Pz23 can be confidently assigned to the PHI-database with an E-value of 1 e-10. With a cutoff of identity >0.5, 39 PHI genes were identified as loss-of-pathogenicity, 271 as reduced virulence, and 82 involved in unaffected pathogenicity in Pz20. For Pz23, 26 PHI genes were found to be associated with loss of pathogenicity, while 252 and 80 genes were linked to reduced virulence and unaffected pathogenicity, respectively. The most prominent PHI genes in both strains are PHI:7143 and PHI:7735, which are associated with reduced virulence. The corresponding proteins in Pz20 and Pz23 are like Cyclophilin-type peptidyl-prolyl cis-trans isomerases (PPIases), which regulate the expression of antioxidant enzymes. Previous studies have demonstrated their involvement in the activity of the secreted virulence factor nuclease (60).

### Horizontal gene transfer analysis

**Fig 5:**
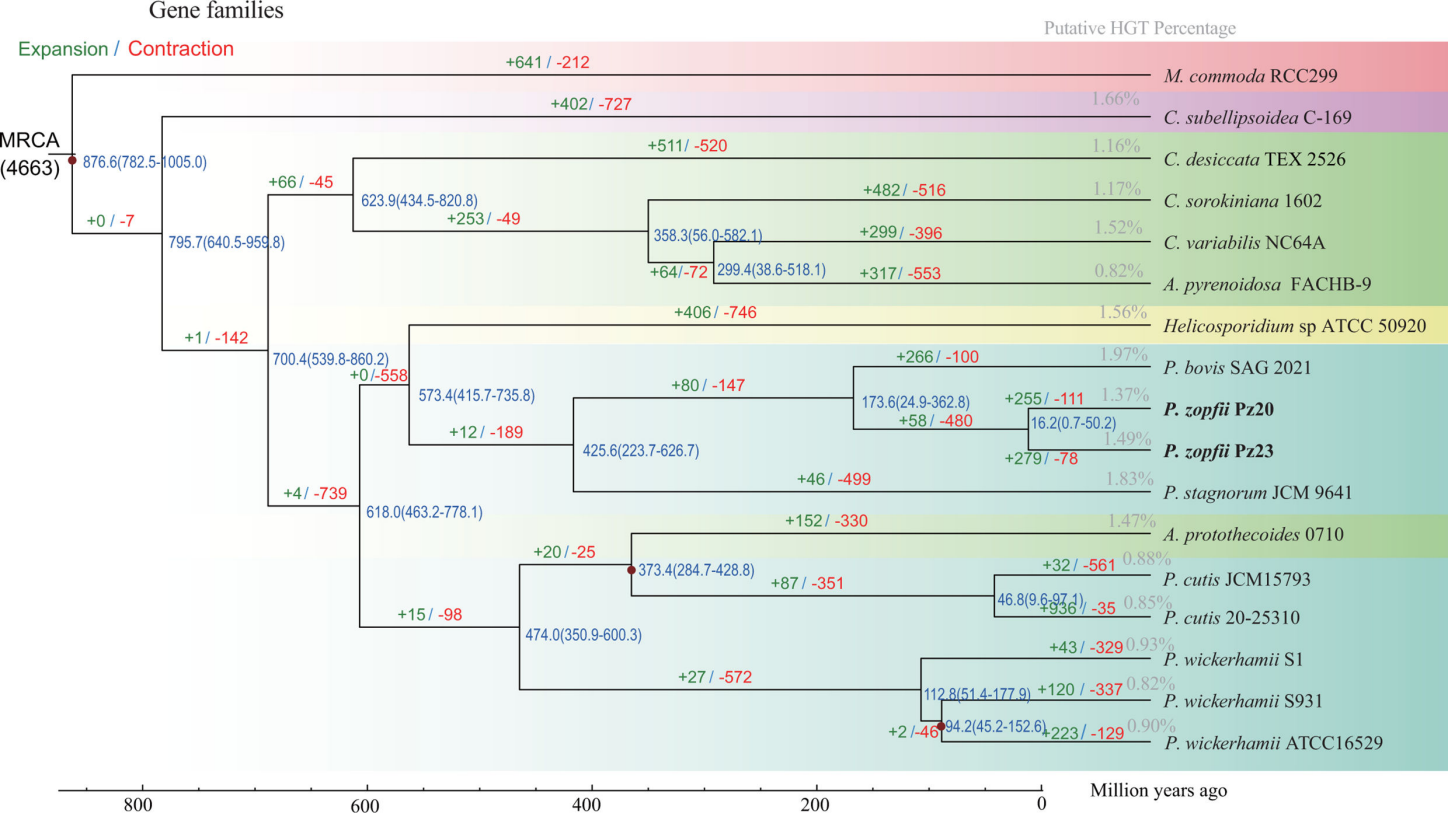
Phylogenetic tree, gene family contraction and expansion, divergence time, and putative HGT percentage among the 17 genomes of green algae.

**Fig 6:**
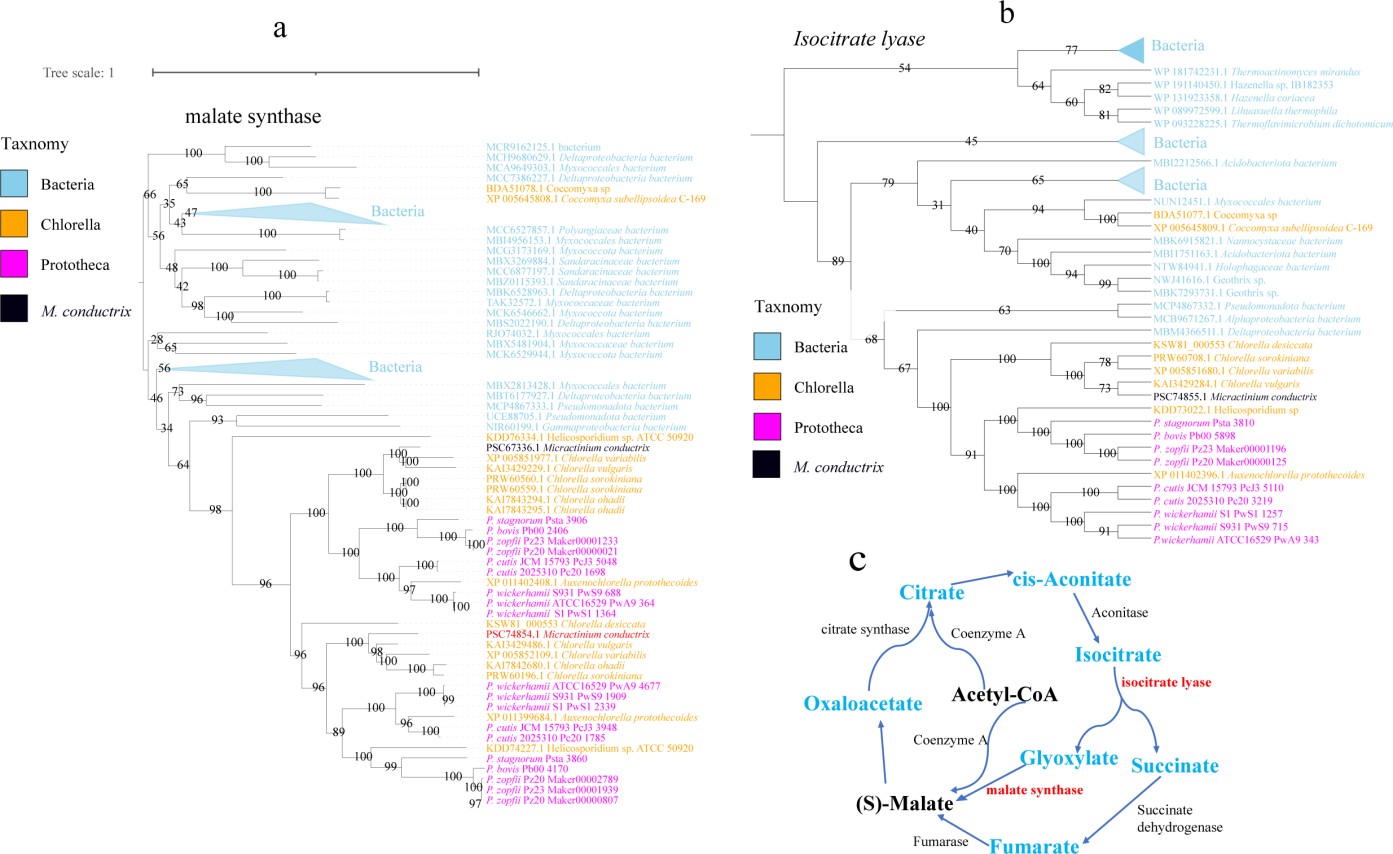
The glyoxylate cycle and related putative horizontal gene transfer events. (a) The phylogenetic tree of putative HGT malate synthase. (b) The phylogenetic tree of putative HGT isocitrate lyase. (c) A diagram of the metabolic pathways of the glyoxylate cycle.

To investigate the potential horizontal gene transfer (HGT) events related to heterotrophs and pathogenicity, all algal species except for the outgroup *M. commoda* were utilized for HGT identification. A total of 47 to 163 genes were identified as putative HGT genes, with the percentage of HGT ranging from 0.82% to 1.97% among the 16 genomes (Table S12). A total of 66 (1.37%) and 73 (1.49%) genes were identified as potential HGT events in Pz20 and Pz23, respectively (Table S12). The percentage of horizontal gene transfer may be associated with the phylogenetic clade of the species, as evidenced by 0.82%–0.93% in the subgroup tree and 1.37%–1.97% in other subgroups (Fig. 5). The percentage of HGT percentage in this study is lower than that reported for diatoms (3%–5%) (24), but slightly higher than previous estimates (~1%) for most species (16). The KEGG enrichment of HGT genes in Pz20 and Pz23 showed that most of gene involved in metabolic pathways including biosynthesis of secondary metabolites, biotin metabolism, carbon metabolism, tryptophan metabolism, and pyruvate metabolism (Fig. S12 and S13). The gene ontology terms for catalytic activity, antioxidant activity, malate synthase activity, glyoxylate cycle, and glyoxylate metabolic process were significantly enriched (*P* < 0.05) in both putative HGT genes of Pz20 and Pz23. These related gene functions from bacterial donors may play important roles in the heterotrophic and pathogenic processes. The HGT of the glyoxylate cycle had been reported as an effective metabolic adaptation strategy for survival and pathogenesis in *Candida glabrata*, an intracellular human fungal pathogen (61). The putative HGT genes were analyzed to identify orthologous groups, and it was found that eight orthologous groups are present in all nine *Prototheca* genomes. Two malate synthase genes from each of the nine strains of *Prototheca* species were horizontally transferred from bacteria (Fig. 6a). Nearly most of transferred malate synthase genes from *Chlorella*, *Prototheca,* and *M. commode* are in the same clade and two genes of *Coccomyxa* in the other group. The malate synthase plays a crucial role in the glyoxylate cycle and contributes to the survival of *Salmonella typhimurium* under conditions of carbon limitation and oxidative stress (62). The identification of an isocitrate lyase, which facilitates the cleavage of isocitrate into succinate and glyoxylate, as having been horizontally transferred from bacteria indicates its potential significance in metabolic evolution (Fig. 6b). Multiple horizontal gene transfer events between various bacterial and eukaryotic lineages have been identified in the phylogenetic trees of malate synthase and isocitrate lyase (63). Malate synthase and isocitrate lyase are indispensable for the glyoxylate cycle (Fig. 6c), which may play a crucial role in carbon and nitrogen metabolism as well as *Prototheca* metabolic evolution and non-photosynthesis. Each of alpha/beta hydrolase (ABH) genes from Pz20 and Pz23 were identified to originate from bacteria (Fig. S14). The gene of monomeric sarcosine oxidase was identified through HGT and found to be homologous to other closely related algae within the same clade, originating from bacteria (Fig. S15). Some other genes, such as NAD(P)/FAD-dependent oxidoreductase, fatty-acid--CoA ligase, estradiol aromatic ring-opening dioxygenase, and peptide chain release factor 1, have also been horizontally transferred from bacteria. These genes are involved in oxidoreductase and fatty acid metabolism, which may play a potential role in adaptation, diversity, or pathogenicity for *Prototheca*.

### Conclusions

Here, we first generated two high-quality genomes of *P. zopfii*, namely strains Pz20 and Pz23, using HiFi long reads sequencing. A total of 74 and 80 contigs were assembled for Pz20 and Pz23, respectively, with genome sizes of 31.2 Mb and 31.3 Mb. The complete plastid genome was assembled with a circular contigs for both strains. The plastids newly generated in this study are the smallest, lacking all photosynthesis-related genes and ATPase. The compact plastid is reduced to one-third the size of its closest related algae. In addition, the transmission from autotrophs to heterotrophs resulted in a significant reduction in nuclear genome size and genes, including carotenoid oxygenase and Pheophorbide an oxygenase, as well as transcription factors (bZIP, FAD-linked oxidase, and helicase), TRs (SET, GNAT, and SNF2), protein kinases, and so on. The lost genes are involved in the processes of cell growth, abiotic stress resistance, host-pathogen interactions, and pathogenicity. Between 0.82% and 1.97% of genes were identified as putative horizontal gene transfers from bacteria, including two pivotal genes involved in the glyoxylate cycle, namely malate synthase and isocitrate lyase. In addition, other HGT genes enriched in KEGG pathways including the biosynthesis of secondary metabolites, biotin metabolism, carbon metabolism, tryptophan metabolism, and pyruvate metabolism. The availability of the two high-quality *P. zopfii* nuclear and plastid genome sequences offers novel insights into the phylogenetic and genomic framework for investigating adaptation, tropic transmission, pathogenicity, and diagnostics.

## MATERIALS AND METHODS

### Sampling, library preparation and sequencing

The *P. zopfii* strains Pz20 and Pz23 were isolated from two patients located in Shanghai and Chengdu, China, respectively. The Pz20 was collected from the infection in a 66-year-old diabetic woman following hand surgery on her middle right finger. The P23 was collected from a 24-year-old woman with a facial tissue infection. The two strains were classified as *P. zopfii* genotype 1, also known as *P. ciferrii* according to the previous protocol and cytb gene (64). The strains were cultivated on Sabouraud dextrose agar medium, incubated at 35°C for 3 days, and subsequently harvested for genome sequencing. The genomic DNA of the two *P. zopfii* strains was extracted using the CTAB method according to a previously established protocol (65) . Each library was generated using 1 µg of input DNA. After shearing into ~300 bp fragments with the Covaris E220 System and performing size selection (300–500 bp), library preparation was carried out using the MGIEasy Universal DNA Library Prep Set (MGI-Tech). 150 bp paired-end reads were sequenced on the MGISEQ-2000 platform. The reads containing adapters were processed to remove PCR duplicates, and subsequently filtered using SOAPnuke version 1.5.3 to eliminate low-quality reads with N bases >1% or quality values ≤10 and low-quality bases >20% (66). The SMRTbell Prep Kit 2.0 was utilized to generate 20 kb single-molecule real-time (SMRT) DNA libraries with barcoding for both strains. The PacBio circular consensus sequencing (CCS) HiFi library was sequenced on the PacBio SequelII platform. The data were split into each strain with barcodes, and the PacBio HiFi reads were refined using SMRTLink v11.0.0, with a minimum read quality threshold of 0.80 and a minimum read length requirement of 7 kb (https://www.pacb.com/support/software-downloads).

To assist the gene annotation, both two strains were cultivated on Sabouraud dextrose agar medium, incubated at 35°C for 3 days and the RNA was isolated from cultured cells using the TRIzol reagent (Invitrogen, USA) according to the manufacturer’s instructions. The library preparation was following the previously study (67) and sequencing on DNBSeq with PE 150.

### Genome survey and genome assembly

Before long-read sequencing, genome size and heterozygosity were estimated for *P. zopfii* Pz20 with the short reads. The 21-mer frequency was calculated for Pz20 using jellyfish (68). The Pz 20 genome was characterized through 21-mer distribution profiling utilizing Genomescope 1.0 (69). Using the long reads, the nuclear genomes of the two *P. zopfii* strains were assembled using hifiasm v0.7 with default parameters (70), demonstrating a high level of proficiency in genome assembly. A complete circular contig was generated for each strain through the utilization of the GetOrganelle pipeline with default parameters (71). The duplicated sequences of heterozygous regions were eliminated by the PurgeHaplotigs pipeline (72). To avoid potential contamination, the assembled contigs underwent Megablast analysis against the GenBank nucleotide (nt) database (as of October 20th, 2022), followed by Minimap2 mapping against the available agal organelle Refseq genome database. Contigs exhibited no bacterial contamination, with only a few organelle sequences detected in contigs that displayed significant differences in GC content compared to the average GC of assembled genome sequences. A complete circular contig of the plastid genome for each *P. zopfii* strain was generated using the GetOrganelle pipeline with default parameters (71).

### Genome annotation

The repeat and gene structures were predicted for the two newly assembled genomes of *P. zopfii*. With the repeat sequence database of RepBase v21.12 (http://www.girinst.org/repbase) (73), the repetitive sequences were identified *via* homology-based methods utilizing RepeatModeler v2.0 (74). (http://www.repeatmasker.org/RepeatModeler/) and LTR_FINDER v1.07 (75) (http://tlife.fudan.edu.cn/ltr_finder/). The repetitive sequences were identified using the ab initio method with RepeatMasker 3.3.0 (76). After identifying repeats, three different algorithms were employed for gene annotation, including ab initio prediction, homology-based annotation, and RNA-Seq data-based annotation.

In the *ab initio* prediction process, the genome sequences that have been masked for repeated elements were utilized to predict coding regions of genes through the utilization of AUGUSTUS v3.2.3 (77) and SNAP (78). In homology-based annotation, a total of 8 published homology protein sequences from *Chlorella variabilis*, *Chlorella sorokiniana*, *Chlorella desiccate*, *Auxenochlorella protothecoides*, three strains of *Prototheca wickerhamii,* and *Coccomyxa subellipsoidea* were integrated with the MAKER pipeline (79). The first-round result of MAKER was used to train a gene model. A total of 7.5 Gb of *P. zopfii* transcriptome sequencing data were generated for RNA-Seq data-based annotation. The RNA reads were respectively aligned to the two *P. zopfii* genome sequences using hista2.2.1 (80). With the aligned results, transcripts were assembled using stringtie2.1.6 (81). The gene prediction and annotation were ultimately obtained through a second round of the MAKER pipeline (79). We obtained 15 algae genome sequences, including those of the genera *Prototheca* and *Auxenochlorella*, from NCBI (National Center for Biotechnology Information) to conduct comparative genomics analysis. The *Prototheca ciferrii* SAG2063 genome was removed due to low (complete BUSCO 53.8% in eukaryote database) quality. Six genomes of *Prototheca ciferrii* SAG2063, *Prototheca bovis* SAG 2021, *Prototheca cutis* 20–25310, *Prototheca cutis* JCM 15793, *Prototheca stagnorum* JCM 9641, *Auxenochlorella protothecoides* 0710, and *Auxenochlorella pyrenoidosa* FACHB-9 had no annotation information available. The six algae genomes were annotated using the same strategies as employed for the *P. zopfii* genome. The plastid genome was annotated utilizing the GeSeq software (82). The genome and gene set were assessed using Benchmarking Universal Single-Copy Orthologs (BUSCO version 5.1.2) against the “chlorophyta_odb10” database, which contains 1519 conserved core eukaryotic genes. The functional annotation of protein-coding genes was conducted in accordance with the methodology previously employed (13).

### Gene family and phylogenetic analysis

The amino acid sequences of the two newly sequenced *P. zopfii* and 14 downloaded Trebouxiophyceae genomes (*Auxenochlorella protothecoides* 0710, *Auxenochlorella pyrenoidosa* FACHB-9, *Chlorella desiccate*, *Chlorella sorokiniana* 1602, *Chlorella variabilis*, *Coccomyxa subellipsoidea* C-169, *P. bovis* SAG 2021, *P. cutis* 2025310, *Prototheca cutis* JCM 15793, *P. stagnorum* JCM 9641, *P. wickerhamii* ATCC16529, *P. wickerhamii* S1, *P. wickerhamii* S931, and *Helicosporidium* sp. ATCC 50920) and outgroup *Micromonas commoda* RCC299 were analyzed. To determine evolutionary relationships among green algae species, the orthologous genes were identified in the clade of the Trebouxiophyceae, and an outgroup *Micromonas commoda* species was examined. First, the orthogroups were clustered using OrthoFinder (v2.3.11) with default parameters (83). The protein sequences from each orthogroup were applied to multiple sequence alignment by MAFFT (v7.310) (84) and trimmed with Trimal-1.4.1 (85) with gappyout model. Then, the alignments were utilized for all the orthologous genes and subjected for phylogenetic tree construction using IQ-TREE (v1.6.12) (86) employing the maximum-likelihood (ML) method and MFP model, with a bootstrap of 1000. The species of *Micromonas commoda* was rooted as an outgroup using TreeBest (https://github.com/Ensembl/treebest). The divergence time of each algal species was identified with the MCMCTree module in PAML (v 4.9, http://abacus.gene.ucl.ac.uk/software/paml.html). Three time-calibrated points [*M.commode–C. reinhardtii* (792.4–1,019.6 Mya), *O. tauri–C. reinhardtii* (781.1–803.1 Mya), and *C. reinhardtii–C. variabilis* (586.9–604.9 Mya)] were derived from the TimeTree database (http://www.timetree.org/), which had been used in previous studies (13). The expansion and contraction analysis of all the algae gene families was performed using CAFÉ (v3.1) (87). To investigate the relationship and patterns of functional diversification in gene domains, we utilized principal components analysis (PCA) to examine the number of InterPro pfam domains (conserved protein domains) across 17 genomes.

### Identification of putative horizontal gene transfer

To identify candidates of horizontally transferred genes, the protein sequences of the 17 target genomes were blasted as a query against the NCBI non-redundant(nr) protein database (20230420) by Diamond (88) with E value = 1e^−10^. With the implementation of this blastp modification, the calculation of AI (Alien Index) was performed using the HGTphyloDetect pipeline (89). The AI with a value of ≥40 and an out_pct value of ≥90% are considered as potential candidates for HGT. In summary, all identified candidate donor species from BLAST searches and phylogenetic analyses were classified into three putative groups: bacteria, archaea, and viruses. The putative candidate genes and targeted donor homolog genes were clustered and analyzed using OrthoFinder (v2.3.11) to identify orthogroups (83). The protein sequences of HGT genes and its related homologs from the Nr databases were subjected to multiple sequence alignment using MAFFT v7.310 (84) and trimmed with Trimal-1.4.1 (85) with gappyout model. The phylogenetic tree was inferred using IQ-TREE multicore version 2.2.0 (86) employing the maximum-likelihood (ML) method and MFP model, with a bootstrap of 1,000. The phylogenies were rooted at intermediate nodes and visualized using iTOL v6 (90). To remove the false positives, for each identified candidate transferred gene, the blast and phylogenetic tree were applied to evaluate its existence as a true gene in the *P. zopfii* genome. If the interested genes were on contigs with a length less than 100 kb, they were excluded from consideration. Candidates of transferred genes were determined after rigorous phylogenetic analyses. Functions of the transferred genes were categorized based on GO and KEGG enrichment annotations (91).

## Data Availability

The data supporting the findings of this study have been deposited into the CNGB Sequence Archive (CNSA) of China National GeneBank DataBase (CNGBdb) (92), with accession number CNP0004538. The data had also been deposited into NCBI under the project number PRJNA1080336. The long-read data for Pz 20 and Pz 23 are, respectively, under the accession SRR28126906 and SRR28126904. The short-read data of Pz 20 was under the accession SRR28126905.

## SUPPLEMENTAL MATERIAL

Supplemental material spectrum.04148-23-s0001.docx Fig. S1 to S15; Tables S1 to S15.

ASM does not own the copyrights to Supplemental Material that may be linked to, or accessed through, an article. The authors have granted ASM a non-exclusive, world-wide license to publish the Supplemental Material files. Please contact the corresponding author directly for reuse.
