# Supplementary material for: Two high-quality Prototheca zopfii genomes provide new insights into their evolution as obligate algal heterotrophs and their pathogenicity
Source: Microbiol Spectr. 2024 Jun 28;12(8):e04148-23. doi: 10.1128/spectrum.04148-23 (PMC11302234; doi:10.1128/spectrum.04148-23)
Supplement: Supplemental material — Fig. S1 to S15; Tables S1 to S15. [file spectrum.04148-23-s0001.docx]

Supplementary materials

**Two** **high quality** ***Prototheca zopfii* genomes provide new insights into their evolution as** **obligate** **algal heterotrophs and** **their pathogenicity**

Jianbo Jian, Zhaoyang Wang, Chunhai Chen, Christopher T. Workman, Xiaodong Fang, Thomas Ostenfeld Larsen, Jian Guo, Eva C. Sonnenschein

Outline

[Figure S1 Genome survey of *Prototheca zopfii* Pz20 using GenomeScope. 2](#_Toc147933801)

[Figure S2 The GC distribution of *P. zopfii* genomes and representative downloaded genomes. 2](#_Toc147933802)

[Figure S3 The repeat divergence plots of denovo and known sequences of *P. zopfii* 3](#_Toc147933803)

[Figure S4 The BUSCO assessment of target 17 green algae genomes. 3](#_Toc147933804)

[Figure S5 Eukaryotic Orthologous Groups (KOG) annotation of *P. zopfii* Pz20 4](#_Toc147933805)

[Figure S6 Eukaryotic Orthologous Groups (KOG) annotation of *P. zopfii* Pz23 4](#_Toc147933806)

[Figure S7 Kyoto Encyclopedia of Genes and Genomes (KEGG) enrichment annotation of *P. zopfii* Pz20 5](#_Toc147933807)

[Figure S8 Kyoto Encyclopedia of Genes and Genomes (KEGG) enrichment annotation of *P. zopfii* Pz23 5](#_Toc147933808)

[Figure S9 The heatmap of InterPro domains (top 10% IPR number) in 17 green algae genomes*.* 6](#_Toc147933809)

[Figure S10 The gene synteny among two *P. zopfii, P. wickerhamii* S1, *P. cutis* JCM15793 and *P. stagnorum.* 6](#_Toc147933810)

[Figure S11 The heatmap of the number of transcript factor of 17 green algae genomes. 7](#_Toc147933811)

[Figure S12 The KEGG enrichment of putative HGT gene in Pz20. 8](#_Toc147933812)

[Figure S13 The KEGG enrichment of putative HGT gene in Pz23. 8](#_Toc147933813)

[Figure S14 The phylogenetic tree of putative HGT alpha/beta hydrolase gene. 9](#_Toc147933814)

[Figure S15 The phylogenetic tree of putative HGT monomeric sarcosine oxidase gene. 9](#_Toc147933815)

[Table S1 Sequencing data statistics of *Prototheca zopfii* genome with DNBSeq platform. 10](#_Toc147933816)

[Table S2 Sequencing data statistics of two strains of *P. zopfii* genomes with PacBio Sequel II platform. 10](#_Toc147933817)

[Table S3. The assembly statistics of two *P. zopfii* genomes. 10](#_Toc147933818)

[Table S4. The detailed information of the 15 downloaded algae genomes. 11](#_Toc147933819)

[Table S5 BUSCO evaluation of two *P. zopfii* genomes. 12](#_Toc147933820)

[Table S6 The plastid statistics information of *P. zopfii* and 9 downloaded Trebouxiophyceae genomes. 12](#_Toc147933821)

[Table S7 Repeat content in assembled *P. zopfii* Pz20 nuclear genomes. 13](#_Toc147933822)

[Table S8 Repeat content in assembled *P. zopfii* Pz23 nuclear genomes. 13](#_Toc147933823)

[Table S9 The comparison of KEGG enrichment annotation in 17 genomes. 14](#_Toc147933824)

[Table S10 Gene functional annotation in two *P. zopfii* genomes. 14](#_Toc147933825)

[Table S11 IPR domains most enriched in *P. zopfii* when compared to other *Prototheca* species. 15](#_Toc147933826)

[Table S12 Statistics of gene families in 17 algae genomes. 16](#_Toc147933827)

[Table S13 The GO enrichment and pathway of contracted and expanded genes. 16](#_Toc147933828)

[Table S14 The putative HGT gene statistics in 16 algae genomes. 17](#_Toc147933829)

[Table S15 The gene ontology enrichment of putative HGT gene statistics in Pz20 and Pz23 genomes. 17](#_Toc147933830)

# Figure S1 Genome survey of *Prototheca zopfii* Pz20 using GenomeScope.

X axes is the coverage (X), y axes is the frequency of 21-mer.

Note: het: heterozygosity; kcov: kmer mean peak; uniq: non-repetitive kmer; observed: the kmer actual distribution by jellyfish analysis; full model: theoretical kmer distribution; unique sequence: non-repetitive kmer; errors: wrong kmer, usually the lower kmer；kmer-peaks: the position of the kmer peak.

# Figure S2 The GC distribution of *P. zopfii* genomes and representative downloaded genomes.

# Figure S3 The repeat divergence plots of denovo and known sequences of *P. zopfii*

# Figure S4 The BUSCO assessment of target 17 green algae genomes.


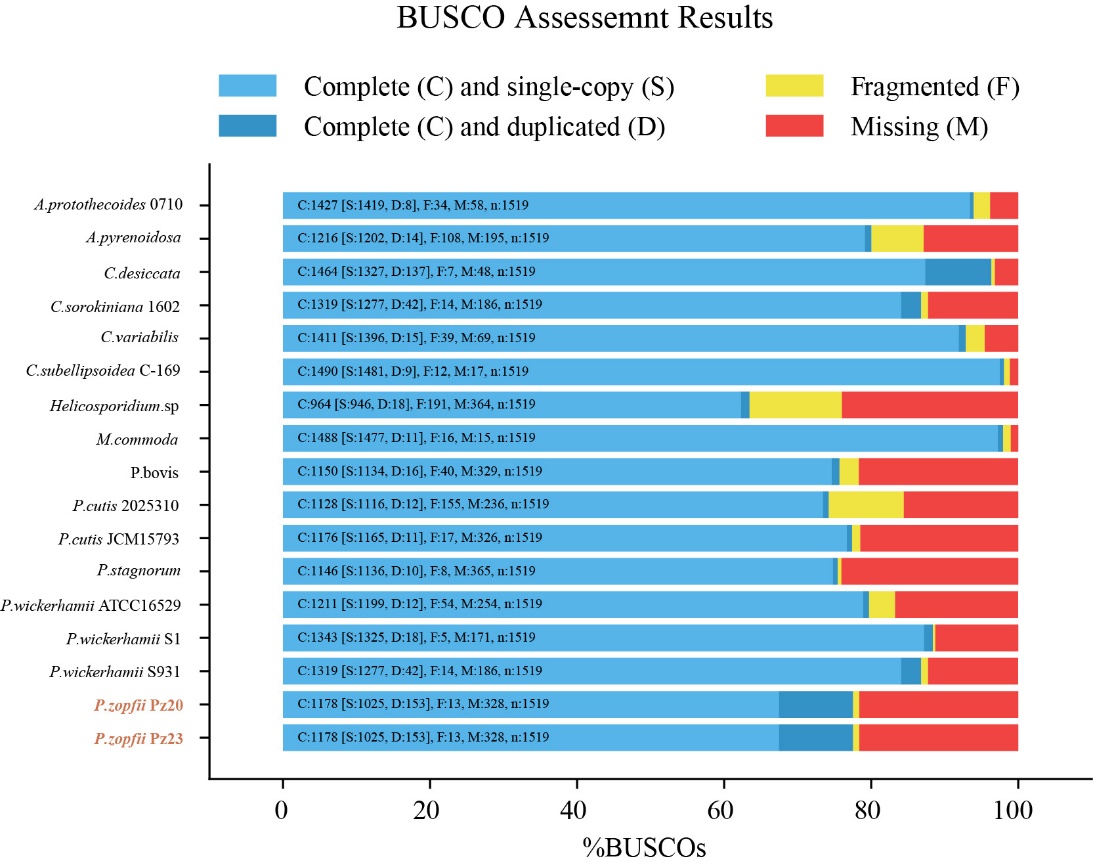


# Figure S5 Eukaryotic Orthologous Groups (KOG) annotation of *P. zopfii* Pz20

# Figure S6 Eukaryotic Orthologous Groups (KOG) annotation of *P. zopfii* Pz23

# Figure S7 Kyoto Encyclopedia of Genes and Genomes (KEGG) enrichment annotation of *P. zopfii* Pz20

# Figure S8 Kyoto Encyclopedia of Genes and Genomes (KEGG) enrichment annotation of *P. zopfii* Pz23

# Figure S9 The heatmap of InterPro domains (top 10% IPR number) in 17 green algae genomes*.*


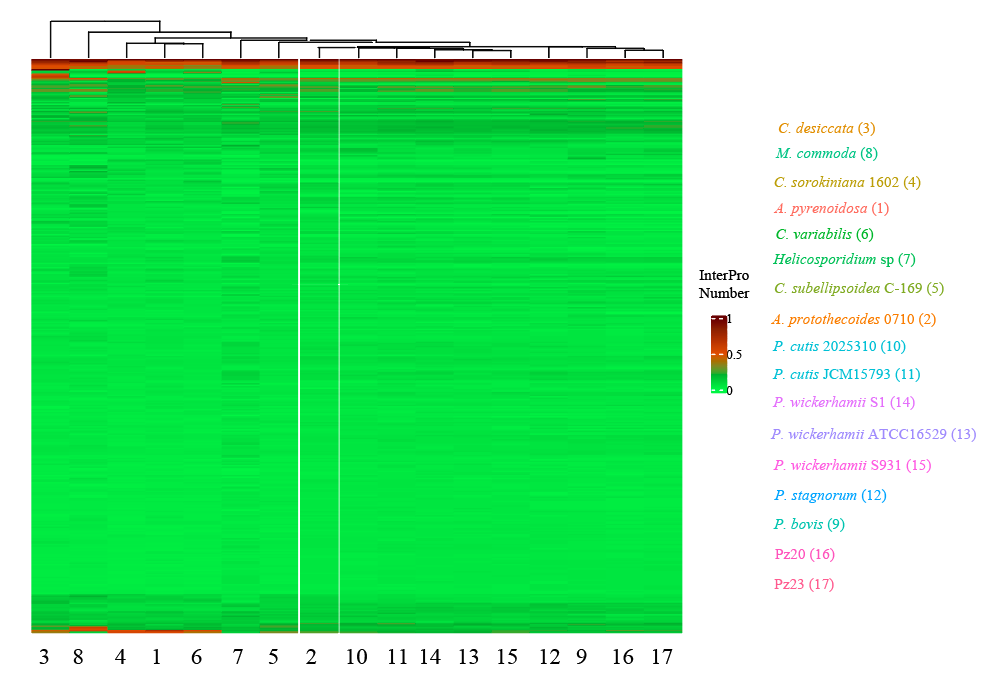


# Figure S10 The gene synteny among two *P. zopfii, P. wickerhamii* S1, *P. cutis* JCM15793 and *P. stagnorum.*

# Figure S11 The heatmap of the number of transcript factor of 17 green algae genomes.

(The green color on the left represents photosynthetic and autotrophic algae, the right represents non-photosynthetic and heterotrophic. *Auxenochlorella pyrenoidosa*: Ap00, *Auxenochlorella protothecoides* 0710:A071, *Chlorella desiccate*:Cdes, *Chlorella sorokiniana* 1602: Cs02, *Coccomyxa subellipsoidea* C169:Csc9, *Chlorella variabilis*:Cv00, *Helicosporidium* sp: Heli, *Micromonas commoda*: Micm, *Prototheca bovis*:Pb00, *Prototheca cutis* 2025310:Pc20, Prototheca cutis JCM15793:Pcjc, *Prototheca stagnorum*:Psta, *Prototheca wickerhamii* S1:Pws1, *Prototheca wickerhamii* S931:Pws9, *P. zopfii* Pz20:Pz20, *P. zopfii* Pz23:Pz23).

# Figure S12 The KEGG enrichment of putative HGT gene in Pz20.

# Figure S13 The KEGG enrichment of putative HGT gene in Pz23.

# Figure S14 The phylogenetic tree of putative HGT alpha/beta hydrolase gene.


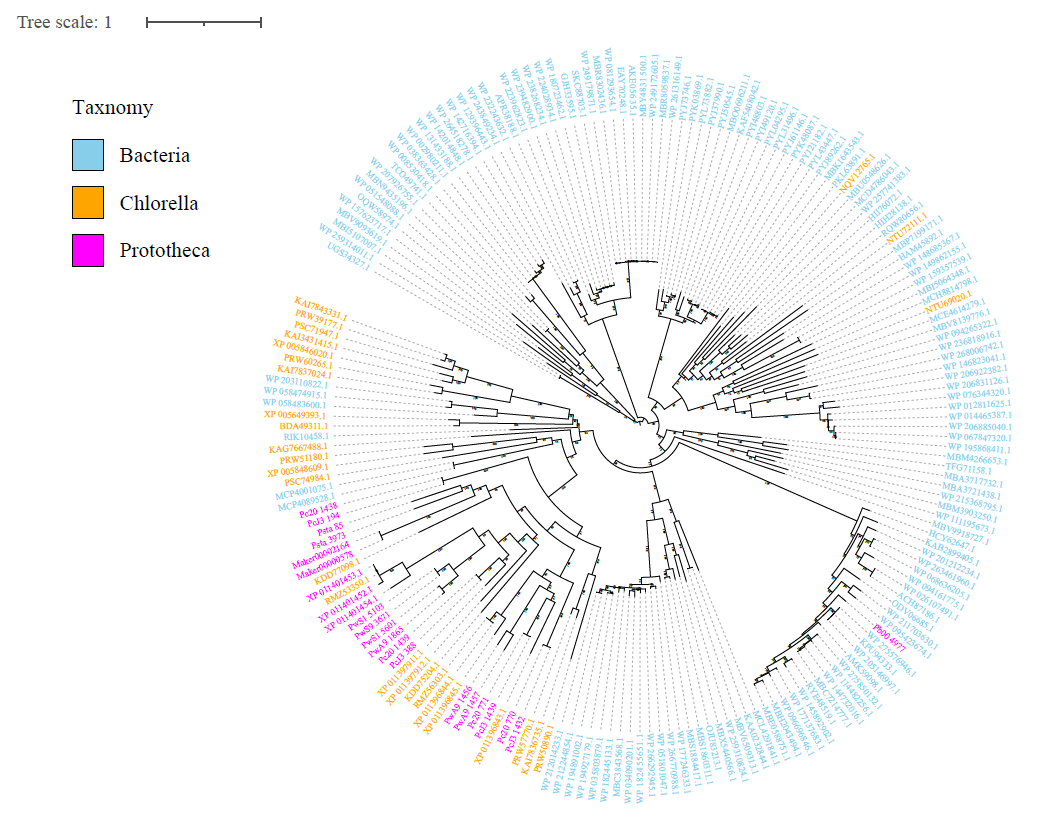


# Figure S15 The phylogenetic tree of putative HGT monomeric sarcosine oxidase gene.


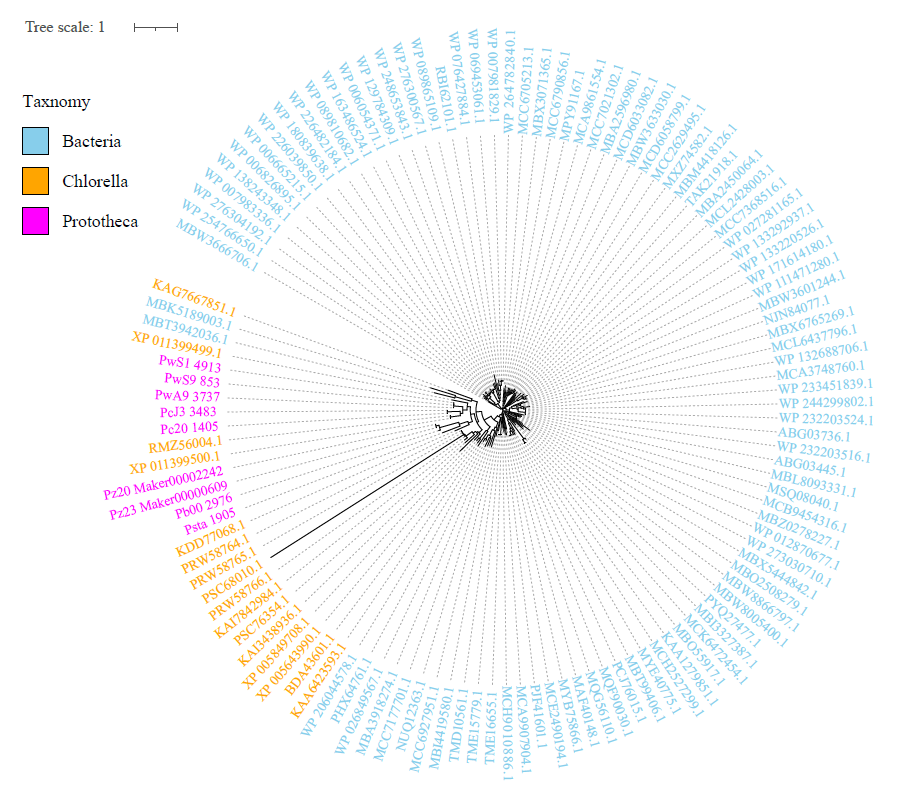


# Table S1 Sequencing data statistics of *Prototheca zopfii* genome with DNBSeq platform.

| Sample | Data | Total number of bases | Q20 | Q30 |
| --- | --- | --- | --- | --- |
| *P. zopfii* Pz 20 | Raw reads(fq1) | 2,398,123,950 | 98.27% | 95.10% |
|  | Clean reads(fq1) | 2,279,922,300 | 98.28% | 95.41% |
|  | Raw reads(fq2) | 2,398,123,950 | 94.71% | 87.47% |
|  | Clean reads(fq2) | 2,279,922,300 | 94.69% | 88.11% |
|  | Clean fq1 and fq2 | 4,559,844,600 | 96.49% | 91.76% |

# Table S2 Sequencing data statistics of two strains of *P. zopfii* genomes with PacBio Sequel II platform.

| **Sample** | **Reads Number** | **Total Length** | **Min Length (reads)** | **Average Length** | **Max Length (reads)** | **Length N50 (reads)** |
| --- | --- | --- | --- | --- | --- | --- |
| Pz20 | 232,658 | 4,117,110,667 | 555 | 17,696 | 37,907 | 17,382 |
| Pz23 | 180,600 | 3,144,938,625 | 531 | 17,414 | 49,713 | 17,078 |

# Table S3. The assembly statistics of two *P. zopfii* genomes.

|  | **Pz20** | | **Pz23** | |
| --- | --- | --- | --- | --- |
|  | **contig** | | **contig** | |
|  | **length(bp)** | **number** | **length(bp)** | **number** |
| Max length | 2,960,522 |  | 2,950,851 |  |
| N10 | 2,501,942 | 2 | 2,179,224 | 2 |
| N20 | 2,155,517 | 3 | 1,829,725 | 3 |
| N30 | 2,148,211 | 4 | 1,783,717 | 5 |
| N40 | 2,039,785 | 6 | 1,579,862 | 7 |
| N50 | 1,987,055 | 7 | 1,261,121 | 9 |
| N60 | 1,787,519 | 9 | 1,159,826 | 11 |
| N70 | 1,572,864 | 11 | 1,043,875 | 14 |
| N80 | 1,294,170 | 13 | 771,190 | 18 |
| N90 | 521,700 | 17 | 203,722 | 25 |
| Total length | 31,193,587 |  | 31,323,842 |  |
| number>2000bp | 74 | | 80 | |
| GC rate | 0.677 | | 0.678 | |

Note: the assembled genomes do not include organelle genomes or contamination sequences.

# Table S4. The detailed information of the 15 downloaded algae genomes.

| **Organism Name** | **Sequencing platform** | **Assembled Size** | **GC%** | **Scaffold Number** | **Contig Number** | **Scaffold N50** | **Contig N50** | **Accession ID** |
| --- | --- | --- | --- | --- | --- | --- | --- | --- |
| *Auxenochlorella protothecoides* 0710 | 454 GS FLX Titanium; Illumina HiSeq 2000 | 22,924,637 | 60.58% | 374 | 1,386 | 285,543 | 35,091 | GCA_000733215.1 |
| *Auxenochlorella pyrenoidosa* FACHB-9 | 454 | 56,992,954 | 56.76% | 1,346 | 6,266 | 1,392,758 | 11,699 | GCA_001430745.1 |
| *Chlorella desiccate* UTEX 2526 | PacBio Sequel | 21,646,774 | 44.93% | 18 | 18 | 1,641,916 | 1,641,916 | GCA_019044685.1 |
| *Chlorella sorokiniana* 1602 | PacBio | 59,566,223 | 63.95% | 159 | 159 | 2,592,956 | 2,592,956 | GCA_002245835.2 |
| *Chlorella variabilis* NC64A | Sanger | 46,159,512 | 61.40% | 414 | 3,957 | 1,469,606 | 27,649 | GCA_000147415.1 |
| *Coccomyxa subellipsoidea* C-169 | Sanger | 48,826,616 | 52.94% | 29 | 29 | 1,959,569 | 1,959,569 | GCA_000258705.1 |
| *Prototheca bovis* SAG 2021 | Illumina MiSeq; 454 | 24,744,895 | 73.53% | 4,555 | 4,555 | 7,940 | 7,940 | GCA_003612995.1 |
| *Prototheca cutis* 2025310 | Illumina MiSeq | 19,388,884 | 61.08% | 5,205 | 5,205 | 5,802 | 5,802 | GCA_016906445.1 |
| *Prototheca cutis* JCM 15793 | HiSeq 2500 | 20,029,045 | 60.24% | 46 | 650 | 1,409,608 | 56,125 | GCA_002897115.2 |
| *Prototheca stagnorum* JCM 9641 | HiSeq 2500 | 16,896,228 | 71.40% | 27 | 853 | 1,107,247 | 33,265 | GCA_002794665.1 |
| *Prototheca wickerhamii* ATCC16529 | PacBio RSII; Illumina MiSeq | 16,700,629 | 62.72% | 19 | 6,578 | 1,578,614 | 5,022 | GCA_016906385.1 |
| *Prototheca wickerhamii* S1 | ONT | 17,573,978 | 64.21% | 19 | 19 | 1,639,047 | 1,639,047 | CNS0462035 |
| *Prototheca wickerhamii* S931 | ONT | 17,453,189 | 64.45% | 26 | 26 | 1,406,360 | 1,406,360 | CNS0462036 |
| *Helicosporidium* sp ATCC 50920 | Illumina HiSeq/ GAIIx; | 12,373,820 | 61.70% | 5,666 | 5,666 | 3,036 | 3,036 | GCA_000690575.1 |
| *Micromonas commoda*  RCC299 | Sanger | 21,109,336 | 63.80% | 19 | 1,394,110 | 1,394,110 | 1,394,110 | GCA_000090985.2 |

# Table S5 BUSCO evaluation of two *P. zopfii* genomes.

| *P. zopfii* | **Type** | **Complete BUSCOs (C)** | **Complete and single-copy BUSCOs (S)** | **Complete and duplicated BUSCOs (D)** | **Fragmented BUSCOs (F)** | **Missing BUSCOs (M)** | **Total BUSCO groups searched** |
| --- | --- | --- | --- | --- | --- | --- | --- |
| Pz20 | Genome BUSCO (number) | 1190 | 1126 | 64 | 25 | 304 | 1519 |
|  | Genome BUSCO (percentage %) | 78.3% | 74.1% | 4.2% | 1.6% | 20.1% | 100.0% |
| Pz23 | Genome BUSCO (number) | 1191 | 1120 | 71 | 26 | 302 | 1519 |
|  | Genome BUSCO (percentage %) | 78.4% | 73.7% | 4.7% | 1.7% | 19.9% | 100.0% |

# Table S6 The plastid statistics information of *P. zopfii* and 9 downloaded Trebouxiophyceae genomes.

| **Species** | **Accession** | **Length (bp)** | **Gene** | **CDS** | **rRNA** | **tRNA** | **GC (plastid)** | **GC (CDS)** |
| --- | --- | --- | --- | --- | --- | --- | --- | --- |
| *C. subellipsoidea* C-169 | NC_015084.1 | 175,731 | 116 | 81 | 3 | 32 | 50.74% | 50.00% |
| *C. variabilis* NC64 | NC_015359.1 | 124,579 | 117 | 82 | 3 | 32 | 34.00% | 35.80% |
| *A. protothecoides* 0710 | NC_023775.1 | 84,576 | 111 | 78 | 2 | 31 | 30.76% | 32.20% |
| *P. cutis* *20-25310* | NC_037480.1 | 51,673 | 72 | 40 | 3 | 29 | 29.69% | 28.30% |
| *P. stagnorum* 20-25310 | NC_037479.1 | 48,188 | 56 | 28 | 3 | 25 | 25.65% | 24.00% |
| *P. wickerhamii* ATCC16529 | NC_054192.1 | 47,997 | 69 | 39 | 1 | 29 | 28.20% | 27.10% |
| *Helicosporidium* sp ATCC 50920 | NC_008100.1 | 37,454 | 54 | 26 | 3 | 25 | 26.92% | 25.00% |
| *P. ciferrii* SAG2063 | NC_037450.1 | 28,698 | 47 | 19 | 3 | 25 | 26.98% | 24.00% |
| *P. bovis* SAG 2021 | NC_045059.1 | 28,638 | 47 | 19 | 3 | 25 | 26.97% | 23.70% |
| *P. zopfii* Pz20 | This study | 28,839 | 76 | 23 | 11 | 42 | 26.98% | 24.50% |
| *P. zopfii* Pz23 | This study | 28,757 | 77 | 23 | 12 | 42 | 26.78% | 24.40% |

# Table S7 Repeat content in assembled *P. zopfii* Pz20 nuclear genomes.

|  | **Repbase TEs** | | **TE protiens** | | **De novo** | | **Combined TEs** | |
| --- | --- | --- | --- | --- | --- | --- | --- | --- |
| **Type** | **Length (Bp)** | **% in genome** | **Length (Bp)** | **% in genome** | **Length (Bp)** | **% in genome** | **Length (Bp)** | **% in genome** |
| DNA | 241,434 | 0.77 | 1,860 | 0.01 | 343 | 0.00 | 243,637 | 0.78 |
| LINE | 109,330 | 0.35 | 969 | 0.00 | 0 | 0.00 | 110,299 | 0.35 |
| SINE | 196,878 | 0.63 | 0 | 0.00 | 101,827 | 0.33 | 295,159 | 0.95 |
| LTR | 138,724 | 0.44 | 186,787 | 0.60 | 2,257,806 | 7.24 | 2,342,686 | 7.51 |
| Other | 0 | 0.00 | 0 | 0.00 | 0 | 0.00 | 0 | 0.00 |
| Unknown | 0 | 0.00 | 0 | 0.00 | 1,286,164 | 4.12 | 1,286,164 | 4.12 |
| Total | 622,531 | 2.00 | 189,616 | 0.61 | 2,391,307 | 11.69 | 3,877,229 | 12.42 |

Note: Repbase TEs: the result of *RepeatMasker* based on Repbase; TE proteins: the result of *RepeatProteinMask* based on Repbase; *De novo*: Result of *RepeatMasker* by using library predicted through *De novo*; Total: combine the results of Repbase TEs, TE proteins and *De novo* with removing the over-lap.

# Table S8 Repeat content in assembled *P. zopfii* Pz23 nuclear genomes.

|  | **Repbase TEs** | | **TE protiens** | | **De novo** | | **Combined TEs** | |
| --- | --- | --- | --- | --- | --- | --- | --- | --- |
| **Type** | **Length (Bp)** | **% in genome** | **Length (Bp)** | **% in genome** | **Length (Bp)** | **% in genome** | **Length (Bp)** | **% in genome** |
| DNA | 222,986 | 0.71 | 5,232 | 0.02 | 30,679 | 0.10 | 255,690 | 0.82 |
| LINE | 102,125 | 0.33 | 2,304 | 0.01 | 0 | 0.00 | 104,429 | 0.33 |
| SINE | 192,616 | 0.61 | 0 | 0.00 | 122,341 | 0.39 | 312,468 | 1.00 |
| LTR | 96,195 | 0.31 | 101,621 | 0.32 | 2,510,828 | 8.02 | 2,599,074 | 8.30 |
| Other | 0 | 0.00 | 0 | 0.00 | 0 | 0.00 | 0 | 0.00 |
| Unknown | 0 | 0.00 | 0 | 0.00 | 1,172,448 | 3.74 | 1,172,448 | 3.74 |
| Total | 557,905 | 1.78 | 109,094 | 0.35 | 3,698,329 | 11.81 | 3,937,916 | 12.57 |

Note: Repbase TEs: the result of *RepeatMasker* based on Repbase; TE proteins: the result of *RepeatProteinMask* based on Repbase; *De novo*: Result of *RepeatMasker* by using library predicted through *De novo*; Total: combine the results of Repbase TEs, TE proteins and *De novo* with removing the over-lap.

# Table S9 The comparison of KEGG enrichment annotation in 17 genomes.

| **Species** | **Environmental adaptation** | **Carbohydrate metabolism** | **Amino acid metabolism** | **Metabolism of terpenoids and polyketides** | **Biosynthesis of other secondary metabolites** | **Energy metabolism** | **Translation** | **Metabolism of cofactors and vitamins** |
| --- | --- | --- | --- | --- | --- | --- | --- | --- |
| *A.pyrenoidosa* | 73 | 460 | 336 | 79 | 66 | 249 | 480 | 287 |
| *A.protothecoides* 0710 | 67 | 355 | 257 | 55 | 40 | 189 | 428 | 218 |
| *C.desiccata* | 82 | 488 | 329 | 121 | 60 | 257 | 548 | 281 |
| *C.sorokiniana* 1602 | 100 | 539 | 378 | 97 | 79 | 281 | 549 | 310 |
| *C.subellipsoidea* C169 | 92 | 482 | 343 | 92 | 87 | 251 | 467 | 301 |
| *C.variabilis* | 99 | 497 | 367 | 88 | 78 | 278 | 491 | 292 |
| *M.commoda* | 142 | 354 | 275 | 88 | 50 | 271 | 489 | 248 |
| *Helicosporidium* sp | 33 | 352 | 267 | 34 | 34 | 130 | 394 | 169 |
| *P.bovis* | 35 | 309 | 228 | 42 | 43 | 132 | 413 | 189 |
| *P.cutis* 2025310 | 86 | 465 | 356 | 50 | 59 | 176 | 555 | 240 |
| *P.cutis* JCM15793 | 70 | 328 | 234 | 35 | 35 | 120 | 393 | 170 |
| *P. stagnorum* | 30 | 255 | 197 | 34 | 51 | 120 | 357 | 164 |
| *P. wickerhamii* ATCC16529 | 65 | 361 | 254 | 40 | 42 | 138 | 424 | 185 |
| *P. wickerhamii* S1 | 55 | 349 | 245 | 39 | 45 | 126 | 396 | 172 |
| *P. wickerhamii* S931 | 53 | 330 | 246 | 34 | 41 | 129 | 407 | 163 |
| Pz20 | 40 | 285 | 221 | 42 | 43 | 137 | 388 | 174 |
| Pz23 | 37 | 288 | 228 | 38 | 42 | 140 | 398 | 175 |

# Table S10 Gene functional annotation in two *P. zopfii* genomes.

| ***P. zopfii*** | **Values** | **Total** | **Nr-**  **Annotated** | **Swissprot-**  **Annotated** | **KEGG-**  **Annotated** | **KOG-**  **Annotated** | **TrEMBL-**  **Annotated** | **Interpro-**  **Annotated** | **GO-**  **Annotated** | **Overall** |
| --- | --- | --- | --- | --- | --- | --- | --- | --- | --- | --- |
| Pz20 | Number | 4,801 | 4,715 | 3,777 | 3,771 | 3,691 | 4,713 | 4,415 | 3,052 | 4,738 |
|  | Percentage | 100% | 98.21% | 78.67% | 78.55% | 76.88% | 98.17% | 91.96% | 63.57% | 98.69% |
| Pz23 | Number | 4,899 | 4,807 | 3,921 | 3,815 | 3,724 | 4,806 | 4,539 | 3,072 | 4,828 |
|  | Percentage | 100% | 98.12% | 80.04% | 77.87% | 76.02% | 98.10% | 92.65% | 62.71% | 98.55% |

# Table S11 IPR domains most enriched in *P. zopfii* when compared to other *Prototheca* species.

| **InterPro ID** | **Description** | **Pb00** | **Pc20** | **PcJC** | **Psta** | **PwA9** | **PwS1** | **PwS9** | **Pz20** | **Pz23** | **Different (>3 domain)** |
| --- | --- | --- | --- | --- | --- | --- | --- | --- | --- | --- | --- |
| IPR006094 | FAD linked oxidase, N-terminal | 8 | 5 | 5 | 4 | 5 | 5 | 5 | 13 | 13 | 7.7 |
| IPR001650 | Helicase, C-terminal | 73 | 74 | 70 | 62 | 66 | 76 | 73 | 77 | 79 | 7.4 |
| IPR007173 | D-arabinono-1,4-lactone oxidase | 7 | 2 | 2 | 3 | 3 | 3 | 3 | 11 | 10 | 7.2 |
| IPR002347 | Short-chain dehydrogenase/reductase SDR | 21 | 15 | 13 | 17 | 10 | 9 | 10 | 19 | 22 | 6.9 |
| IPR020683 | Ankyrin repeat-containing domain | 15 | 19 | 16 | 17 | 17 | 15 | 21 | 25 | 20 | 5.4 |
| IPR001155 | NADH:flavin oxidoreductase/NADH oxidase, N-terminal | 4 | 1 | 1 | 5 | 1 | 1 | 2 | 6 | 7 | 4.4 |
| IPR029481 | ABC-transporter N-terminal domain | 3 | 1 | 1 | 0 | 0 | 0 | 0 | 3 | 6 | 3.8 |
| IPR013057 | Amino acid transporter, transmembrane domain | 27 | 28 | 20 | 19 | 20 | 19 | 20 | 26 | 25 | 3.6 |
| IPR008758 | Peptidase S28 | 7 | 3 | 1 | 1 | 2 | 1 | 2 | 7 | 5 | 3.6 |
| IPR006139 | D-isomer specific 2-hydroxyacid dehydrogenase, catalytic domain | 7 | 4 | 3 | 3 | 3 | 4 | 4 | 8 | 7 | 3.5 |
| IPR006073 | GTP binding domain | 17 | 18 | 16 | 14 | 15 | 18 | 18 | 20 | 20 | 3.4 |
| IPR025714 | Methyltransferase domain | 3 | 0 | 1 | 1 | 1 | 1 | 1 | 4 | 5 | 3.4 |
| IPR006845 | Pex, N-terminal | 3 | 3 | 2 | 2 | 3 | 3 | 3 | 6 | 6 | 3.3 |
| IPR011545 | DEAD/DEAH box helicase domain | 50 | 51 | 49 | 42 | 45 | 53 | 52 | 51 | 53 | 3.1 |
| IPR023370 | YaeB-like, N-terminal domain | 1 | 1 | 1 | 0 | 1 | 1 | 1 | 4 | 4 | 3.1 |

The disparity in mean values between two *P. zopfii* strains and other seven Prototheca species with a numerical domain greater than three.

# Table S12 Statistics of gene families in 17 algae genomes.

| **Species** | **Number of genes** | **Number of genes in orthogroups** | **Number of unassigned genes** | **Percentage of genes in orthogroups** | **Number of species-specific orthogroups** | **Number of genes in species-specific orthogroups** | **Percentage of genes in species-specific orthogroups** |
| --- | --- | --- | --- | --- | --- | --- | --- |
| Ap00 | 8,576 | 8,263 | 313 | 96 | 14 | 40 | 0.5 |
| Ap71 | 7,013 | 6,188 | 825 | 88 | 25 | 106 | 1.5 |
| Cdes | 9,551 | 8,832 | 719 | 93 | 200 | 899 | 9.4 |
| Cs02 | 10,383 | 9,678 | 705 | 93 | 195 | 528 | 5.1 |
| Csc9 | 9,838 | 7,998 | 1,840 | 81 | 238 | 905 | 9.2 |
| Cv00 | 9,779 | 9,237 | 542 | 95 | 79 | 238 | 2.4 |
| Heli | 6,033 | 5,128 | 905 | 85 | 28 | 150 | 2.5 |
| Micm | 10,137 | 6,991 | 3,146 | 69 | 282 | 882 | 8.7 |
| Pb00 | 6,804 | 5,863 | 941 | 86 | 50 | 251 | 3.7 |
| Pc20 | 8,541 | 7,908 | 633 | 93 | 8 | 23 | 0.3 |
| Pcjc | 5,596 | 5,547 | 49 | 99 | 3 | 13 | 0.2 |
| Psta | 4,909 | 4,646 | 263 | 95 | 8 | 34 | 0.7 |
| Pwa9 | 6,079 | 5,967 | 112 | 98 | 3 | 8 | 0.1 |
| Pws1 | 5,693 | 5,509 | 184 | 97 | 1 | 2 | 0 |
| Pws9 | 5,703 | 5,529 | 174 | 97 | 5 | 14 | 0.2 |
| Pz20 | 4,801 | 4,791 | 10 | 99.8 | 8 | 130 | 2.7 |
| Pz23 | 4,899 | 4,768 | 131 | 97 | 8 | 64 | 1.3 |

(*Auxenochlorella pyrenoidosa*: Ap00, *Auxenochlorella protothecoides* 0710: A071, *Chlorella desiccate*:Cdes, *Chlorella sorokiniana* 1602: Cs02, *Coccomyxa subellipsoidea* C169:Csc9, *Chlorella variabilis*:Cv00, *Helicosporidium* sp: Heli, *Micromonas commoda*: Micm, *Prototheca bovis*:Pb00, *Prototheca cutis* 2025310:Pcjc, *Prototheca stagnorum*:Psta, *Prototheca wickerhamii* S1:Pws1, *Prototheca wickerhamii* S931:Pws9, *P. zopfii* Pz20:Pz20, *P. zopfii* Pz23:Pz23).

# Table S13 The GO enrichment and pathway of contracted and expanded genes.

| The contracted or expanded genes in the clade of the heterotrophs | | |  |  |
| --- | --- | --- | --- | --- |
| GO function | Gene number | GO |  |  |
| isomerase activity | 4 | GO:0003755 |  |  |
| protein kinase activity | 4 | GO:0004672 |  |  |
| Pathway | Gene number | Pvalue | Qvalue | Pathway ID |
| Plant hormone signal transduction | 3 | 0.000193912 | 0.00096956 | ko04075 |
| MAPK signaling pathway-plant | 3 | 0.000743548 | 0.00185887 | ko04016 |
| The expanded gene Pathway in the *P. zopfii* branch |  |  |  |  |
| #Pathway | | Gene number | Pvalue | Qvalue |
| Ascorbate and aldarate metabolism | 11 | 2.00E-17 | 8.01E-17 | ko00053 |
| Biosynthesis of secondary metabolites | 9 | 0.005077299 | 1.02E-02 | ko01110 |

# Table S14 The putative HGT gene statistics in 16 algae genomes.

| Species | Gene number | HGT | Percent |
| --- | --- | --- | --- |
| *C. subellipsoidea* C-169 | 9,838 | 163 | 1.66% |
| *C. desiccata* | 9,551 | 111 | 1.16% |
| *C. sorokiniana* 1602 | 10,383 | 122 | 1.17% |
| *C. variabilis* | 9,779 | 149 | 1.52% |
| *A. pyrenoidosa* | 8,576 | 70 | 0.82% |
| *Helicosporidium* sp | 6,033 | 94 | 1.56% |
| *P. bovis* | 6,804 | 134 | 1.97% |
| *P. zopfii* Pz20 | 4,801 | 66 | 1.37% |
| *P. zopfii* Pz23 | 4,899 | 73 | 1.49% |
| *P. stagnorum* | 4,909 | 90 | 1.83% |
| *A. protothecoides* 0710 | 7,013 | 103 | 1.47% |
| *P. cutis* JCM 15793 | 5,596 | 49 | 0.88% |
| *P. cutis* 2025310 | 8,541 | 73 | 0.85% |
| *P. wickerhamii* S1 | 5,693 | 53 | 0.93% |
| *P. wickerhamii* S931 | 5,703 | 47 | 0.82% |
| *P.wickerhamii* ATCC16529 | 6,079 | 55 | 0.90% |

# Table S15 The gene ontology enrichment of putative HGT gene statistics in Pz20 and Pz23 genomes.

| **Species** | **Gene Ontology** **term** | **Cluster frequency** | **Genome frequency of use** | **Corrected P-value** |
| --- | --- | --- | --- | --- |
| Pz20 | catalytic activity | 37 out of 41 genes, 90.2% | 1470 out of 2563 genes, 57.4% | 0.00019 |
|  | antioxidant activity | 4 out of 41 genes, 9.8% | 16 out of 2563 genes, 0.6% | 0.00455 |
|  | malate synthase activity | 2 out of 41 genes, 4.9% | 2 out of 2563 genes, 0.1% | 0.01273 |
|  | pyrroline-5-carboxylate reductase activity | 2 out of 41 genes, 4.9% | 2 out of 2563 genes, 0.1% | 0.01273 |
|  | oxidoreductase activity | 13 out of 41 genes, 31.7% | 297 out of 2563 genes, 11.6% | 0.02299 |
|  | glyoxylate cycle | 2 out of 31 genes, 6.5% | 2 out of 1917 genes, 0.1% | 0.02278 |
|  | glyoxylate metabolic process | 2 out of 31 genes, 6.5% | 2 out of 1917 genes, 0.1% | 0.02278 |
| Pz23 | catalytic activity | 34 out of 37 genes, 91.9% | 1465 out of 2545 genes, 57.6% | 0.00016 |
|  | malate synthase activity | 2 out of 37 genes, 5.4% | 3 out of 2545 genes, 0.1% | 0.02384 |
|  | oxidoreductase activity | 12 out of 37 genes, 32.4% | 306 out of 2545 genes, 12.0% | 0.03252 |
|  | glyoxylate cycle | 2 out of 25 genes, 8.0% | 3 out of 1920 genes, 0.2% | 0.02471 |
|  | glyoxylate metabolic process | 2 out of 25 genes, 8.0% | 3 out of 1920 genes, 0.2% | 0.02471 |
